# Neuroinflammation in Alzheimer’s and Parkinson’s diseases: pathogenic mechanisms and therapeutic strategies

**DOI:** 10.1186/s40035-026-00571-3

**Published:** 2026-07-29

**Authors:** Qin-qin Wang, Qing-qing Sun, Yong-shun Guo, Shu Yin, Jia-wei Zhou

**Affiliations:** 1 School of Mental Health, Jining Medical University, Jining, China; 2 Key Laboratory of Medicinal Resources and Natural Pharmaceutical Chemistry, The Ministry of Education; College of Life Sciences, Shaanxi Normal University, Xi’an, China; 3 Institute of Neuroscience, CAS Center for Excellence in Brain Science and Intelligence Technology, Chinese Academy of Sciences, Shanghai, 200031 China; 4 Distinctive College of Health Social Services and Development, Department of Social Work and Health Management (Mental Health Education Center), Xihua University, Chengdu, 610039 China; 5 School of Future Technology, University of Chinese Academy of Sciences, Beijing, 100049 China

**Keywords:** Neuroinflammation, Alzheimer’s disease, Parkinson’s disease, Glial cells, Advanced methodologies, Therapeutic strategies

## Abstract

Neuroinflammation is increasingly recognized as a key contributor and amplifier associated with the pathogenesis of Alzheimer’s disease (AD) and Parkinson’s disease (PD). Neuroinflammation occurs throughout various stages of these diseases with expanding complexity. Currently, no effective therapies exist that specifically target neuroinflammatory processes in these disorders. In this review, we synthesize current understanding of central and peripheral inflammatory mechanisms implicated in both diseases. We illustrate how endogenous pathological triggers, such as amyloid-β (Aβ) peptide, hyperphosphorylated tau, and α-synuclein, activate glial cells, contributing to chronic neuroinflammation that exacerbates neurodegeneration. Additionally, peripheral factors, including systemic inflammation, environmental exposures, and gut-brain axis interactions, are discussed for their roles in modulating neuroinflammatory responses. Notably, the underappreciated roles of oligodendrocyte precursor cells and oligodendrocytes in neuroimmune crosstalk are also highlighted. Advanced methodologies, including glial cell imaging, single-cell transcriptomics, and human induced pluripotent stem cell-derived organoid models, are providing unprecedented insights into the molecular and cellular mechanisms underlying neuroinflammation. Finally, we evaluate emerging therapeutic strategies and ongoing clinical trials targeting neuroinflammatory pathways and analyze the potential of immunomodulatory approaches to slow disease progression. This comprehensive review emphasizes that precise targeting of neuroinflammation represents a tractable strategy for developing effective disease‑modifying treatments for AD and PD.

## Background

Alzheimer’s disease (AD) and Parkinson’s disease (PD) are the most prevalent neurodegenerative disorders worldwide  [1, 2]. Pathologically, AD is characterized by extracellular deposits of amyloid-β (Aβ) forming senile plaques, and intracellular neurofibrillary tangles composed of hyperphosphorylated tau protein  [1]. In contrast, PD is classically characterized by the progressive degeneration of dopaminergic (DA) neurons in the substantia nigra pars compacta (SNpc), accompanied by accumulation of aggregated α-synuclein, a hallmark closely associated with both motor and non-motor manifestations of the disease  [2, 3]. The incidence of both disorders increases substantially with aging, placing mounting pressure on the global health care system  [4, 5]. With disease progression, a proportion of AD and PD patients develop cognitive deficits that can severely impair everyday functioning  [6, 7]. Consequently, deciphering the underlying pathogenic pathways and identifying therapeutic targets have emerged as pressing priorities in contemporary neuroscience research.

Although the exact molecular and cellular drivers of AD and PD remain incompletely elucidated, accumulating evidence highlights several pivotal contributors, including genetic susceptibility, exposure to environmental toxins, and oxidative stress, all of which perturb neuronal homeostasis and promote  pathogenic cascades  [8–10]. Of these factors, neuroinflammation has emerged as a vital element in the pathophysiology of both disorders  [1, 11]. Clinical and preclinical data indicate that neuroinflammation is strongly associated with accelerated disease progression in AD and PD [12–14]. However, whether it serves as a primary pathogenic initiator remains to be further validated and clarified. Beyond inflammation orchestrated by central glial cells, which are principal immune effectors within the brain, peripheral inflammatory processes also play a significant role in fostering and amplifying neuroinflammation in these diseases  [15–17]. The bidirectional communication between central and peripheral immune compartments adds further complexity to the pathobiological landscape of AD and PD. Consequently, gaining mechanistic insights into neuroinflammation is imperative for the rational design of immunomodulatory strategies to mitigate disease progression.

In this review, we synthesize current advances in the role of neuroinflammation in the onset and progression of AD and PD, as well as promising intervention strategies, building upon our earlier work on neuroinflammation published in 2015  [18]. Research over the past decade has yielded unprecedented insights. Notably, the long-standing view of microglia as strictly polarized M1/M2 subtypes has been supplanted by more nuanced models. The classification of astrocytes into neurotoxic A1 and neuroprotective A2 phenotypes [19] has been refuted in a major consensus article, as this oversimplified paradigm fails to accurately capture their dynamic functional state transitions in response to central nervous system (CNS) insults [20]. Furthermore, recognition of the body–brain axis has revealed a systemic dimension of neuroinflammation, reshaping our conceptualization of its global regulatory landscape. Methodologically, cutting-edge technologies, most prominently single-cell RNA sequencing (scRNA-seq), have enabled detailed characterization of gene expression at the individual cell level. Motivated by these transformative developments, we examine the development and propagation of neuroinflammation, the principal cellular sensors and signaling pathways involved, and the complex intercellular crosstalk within the neuroimmune network. We also highlight innovative methodological breakthroughs and emerging immunomodulatory therapies targeting neuroinflammation. By synthesizing these developments, we aim to establish an integrated conceptual framework for deciphering AD and PD pathogenesis and to outline future directions for the development of effective treatments against these devastating disorders.

## Neuroinflammation in AD and PD

Neuroinflammation denotes the inflammatory reactions occurring within the CNS in response to diverse pathological stimuli, such as infectious agents or toxic exposures. These responses are principally orchestrated by glial cells—most notably microglia and astrocytes—which release pro-inflammatory cytokines (e.g., interleukin [IL]-1β, tumor necrosis factor-α [TNF-α]), chemokines (e.g., C-X-C motif chemokine ligand 1), and reactive oxygen species. Under conditions of blood–brain barrier (BBB) compromise, capillary endothelial cells and infiltrating peripheral immune cells can further intensify the inflammatory cascade, thereby amplifying the CNS immune activation  [21].

### CNS triggers of neuroinflammation

**Fig. 1:**
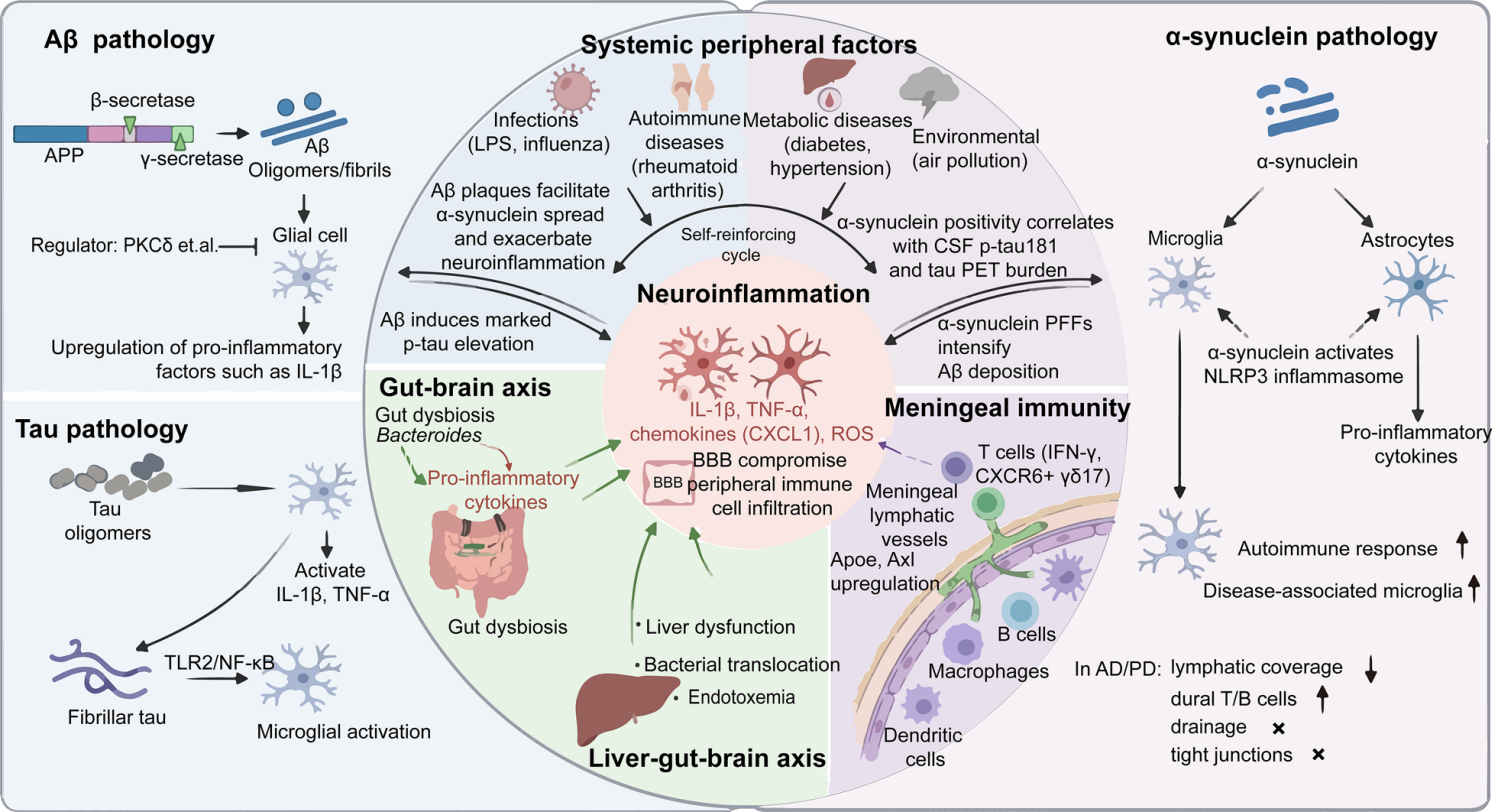
Triggers of neuroinflammation in AD and PD. Neuroinflammation is modulated by multiple internal and external factors. Intrinsic factors, such as pathological proteins (Aβ, tau, α-synuclein) represent important internal triggers contributing to neuroinflammation associated with AD and PD. These proteins show interactions across disease contexts, with underlying molecular mechanisms remaining to be fully clarified. External factors include systemic peripheral factors, such as environmental elements, infections, autoimmune diseases, and metabolic disorders, as well as peripheral organ-brain axes (e.g., gut-brain axis), all of which are linked to neuroinflammation in AD and PD

Aβ peptides are produced from the sequential proteolytic processing of amyloid precursor protein (APP) by β- and γ-secretases  [22]. In vitro, Aβ fibrils upregulate inflammatory chemokines and cytokines, such as IL-1β and IL-6, in astrocytoma cell line, thereby contributing to the activation of neuroinflammatory signaling  [23] (Fig. 1). In vivo, 5 × FAD mice, a transgenic model exhibiting AD-like Aβ deposition, display heightened microglial activation, and this response is diminished by PCSK9 (proprotein convertase subtilisin/kexin type 9) ablation  [23]. Likewise, APP/PS1 mice, another widely used AD model, exhibit activation of both astrocytes and microglia, together with elevated levels of pro-inflammatory cytokines, including IL-1β and TNF-α  [24]. BV2 microglial cells exposed to lipopolysaccharide (LPS) and Aβ exhibit robust expression of inflammatory mediators such as IL-1β  [24]. Oligomeric Aβ markedly enhances the production of pro-inflammatory factors in BV-2 microglial cells, and this effect is mitigated by inhibition of protein kinase C-δ (Fig. 1), which also attenuates microglia-related neuroinflammation in APPswe/PS1dE9 mice  [25]. In addition, transplantation of “Aβ oligomer-primed” microglia into wild-type mice elicits synucleinopathy and tauopathy, accompanied by neurodegeneration  [26]. Collectively, these findings position Aβ-associated neuroinflammation as a pivotal molecular nexus potentially linking multiple proteinopathies within the CNS.

Abnormal hyperphosphorylation of tau protein is another core pathological feature in AD. As a microtubule-associated protein, tau is essential for microtubule stability, neurite outgrowth, and maintenance of neuronal polarity  [27, 28]. Neuroinflammatory glial activation is prominently observed in PS19 mice expressing the human P301S tau. This response is attenuated in some brain regions upon forebrain deletion of BIN1 (bridging integrator 1)  [29]. In the tau-P301S tauopathy model, increased activation of both astrocytes and microglia parallels intensified neuroinflammatory signaling. Tau oligomers stimulate primary microglia to produce elevated levels of pro-inflammatory cytokines, such as IL-1β and TNF-α  [30] (Fig. 1). Likewise, fibrillar tau triggers microglial activation and upregulates expression of inflammatory cytokines, an effect potentially mediated through Toll-like receptor 2 (TLR2)/nuclear factor-κB (NF-κB) signaling  [31] (Fig. 1). Besides, tau-induced generation of double-stranded RNA has been identified as a driver of innate immune activation in the *Drosophila melanogaster* brain [32].

In PD, neuroinflammation is tightly intertwined with pathological protein accumulation, with α-synuclein aggregation serving as a principal stimulus. α-Synuclein is largely concentrated in presynaptic nerve terminals and in discrete domains of the nuclear envelope  [33]. In the A53T α-synuclein transgenic mouse model of PD, pronounced activation of astrocytes and microglia is observed across multiple brain regions, including the cerebral cortex and substantia nigra (SN)  [34]. α-Synuclein contributes to the activation of microglia, accompanied by enhanced pro-inflammatory signaling via pathways such as activation of the NOD-like receptor family pyrin domain containing 3 (NLRP3) inflammasome [35–38]. More recently, α-synuclein has been shown to elicit an autoimmune response that promotes microglial activation and upregulates expression of pro-inflammatory factors (Fig. 1), and induce the A1 astrocytic phenotype  [39]. Together, these findings highlight the potent pro-inflammatory effects of α-synuclein on glial cells in PD pathogenesis.

Collectively, the hallmark pathological proteins Aβ, tau, and α-synuclein serve as key endogenous factors associated with neuroinflammation. Chronic neuroinflammation can in turn promote the aggregation and propagation of these proteins  [27, 40], supporting a self-reinforcing cycle between proteinopathy and neuroinflammation that amplifies pathology. Nevertheless, the precise mechanisms underlying the roles of these proteins across distinct disease stages remain to be fully clarified.

In addition, these pathological proteins are not confined to a single disorder, but rather intersect across multiple neurodegenerative conditions. For instance, α-synuclein aggregates have been documented in AD brains  [41], and α-synuclein positivity correlates with increased cerebrospinal fluid (CSF) level of phosphorylated tau181 and regional tau burden measured by positron emission tomography (PET) in brain regions typically affected in AD  [42] (Fig. 1). Conversely, Aβ deposition also occurs in PD  [43], and aberrant tau phosphorylation has been indicated in PD and linked to DA neuron degeneration (Fig. 1), although the majority of tau aggregates do not colocalize with α-synuclein aggregates. Intriguingly, pathological tau may appear early in mild motor deficit (MMD) cases irrespective of the Lewy pathology, with comparable tauopathy severity between MMD and MMD-with-nigral Lewy bodies (MMD-LB). In contrast, the tau burden is lower in typical PD and rarely detected in the nigrostriatal system in advanced stages of PD, suggesting that abnormal tau may represent an early pathogenic event in PD  [44].

Animal studies further revealed cross-talks among these proteins: Aβ plaques facilitate the spread and accumulation of α-synuclein and exacerbate neuroinflammation [45, 46] (Fig. 1). Aβ also induces marked elevations in p-tau  [47]. α-Synuclein preformed fibrils intensify Aβ deposition in APPswe/PS1dE9 mice  [46]. Together, these data underscore that Aβ, tau, and α-synuclein are not strictly disease-specific, but engaged in intricate, trans-pathological interactions, contributing to inflammation amplification (Fig. 1).

The role of the meninges in the maintenance and regulation of CNS immunity has attracted much attention these years. The meninges, positioned at the interface between the periphery and the brain, are continuous membranous layers encasing the CNS parenchyma and serve as a critical conduit for crosstalk between these compartments  [48]. Meningeal immunity encompasses a heterogeneous repertoire of immune cells, including T lymphocytes, B lymphocytes, dendritic cells, neutrophils, and mast cells, which collectively coordinate both innate and adaptive immune responses  [48–50]. This cellular landscape is complemented by a dynamic stromal network, encompassing permeable vasculature, lymphatics, and diverse cytokine signaling pathways, that orchestrates CNS homeostasis. [51]. Together, these components are essential for immune surveillance, while also contributing to the preservation of cognitive and behavioral functions  [52–54].

The dural meningeal αβ T cells produce interferon-γ (IFN-γ) and the γδ17 T cells express high levels of CXCR6, implicating chemokine-mediated recruitment and activation in this tissue  [50, 54, 55]. Meningeal macrophages confer protection against viral neuroinfections  [56], and enhancement of Th17 cell drainage through the meningeal lymphatic vessels has been demonstrated to alleviate neuroinflammation following subarachnoid hemorrhage  [57]. These findings highlight the indispensable role of the meningeal immune system in shaping the neuroinflammatory processes.

Emerging evidence further links meningeal immunity to the pathogenesis of neurodegenerative diseases, such as AD and PD. Mouse AD models exhibit reduced meningeal lymphatic vessel coverage, increased numbers of dural T and B cells, and vascular deposition of Aβ within the meninges  [58, 59] (Fig. 1). Transcriptomic analyses revealed that the lymphatic dysfunction in 5× FAD mice upregulates expression of microglial activation-associated genes, such as *Apoe* and *Axl*, driving microglia from a homeostatic to a disease-associated state  [58, 60, 61] (Fig. 1). In PD, studies using α-synuclein preformed fibril models showed that the pathological onset is followed by delayed meningeal lymphatic drainage, disruption of lymphatic endothelial tight junctions, and intensified meningeal inflammation  [62] (Fig. 1). Moreover, in transgenic mice overexpressing mutant α-synuclein, obstruction of meningeal lymphatic outflow exacerbates the PD-related pathology [63]. Collectively, these observations demonstrate that meningeal immunity is deeply embedded in the pathogenesis of AD and PD, likely through mechanisms that involve dysregulation of glial function and perpetuation of the neuroinflammatory cascades (Fig. 1).

Several critical questions remain unanswered. For example, how do the aggregation patterns of these proteins differ between AD and PD? How do their effects on the initiation and progression of neuroinflammation diverge? Does the α-synuclein-mediated microglial activation promote Aβ or tau pathology? Addressing these issues requires identification of disease-specific pathways through which these proteins modulate neuroinflammation.

### Peripheral triggers of neuroinflammation

Peripheral and central inflammatory processes are intimately interconnected. In the Tg197 transgenic mouse model with peripheral overexpression of TNF-α, scRNA-seq identified a subpopulation of inflammatory macrophages exhibiting markers reminiscent of activated microglia, which displayed altered gene expression, enriched for transcripts linked to cytokines and complement factors  [64]. This observation provides direct evidence that systemic inflammation is linked with altered immune homeostasis within the CNS. Given that neuroinflammation is a key contributor to neurodegeneration  [1, 65], peripheral factors capable of modulating inflammatory activity may also influence disease progression.

#### Systemic peripheral factors driving neuroinflammation in AD and PD

Mounting evidence highlights the significant contribution of peripheral factors to neuroinflammation in AD and PD. These factors encompass environmental exposures, infections, autoimmune conditions, and metabolic disorders—all implicated in the pathogenesis of both diseases  [66, 67] (Fig. 1). For example, systemic administration of LPS induces microglial activation and exacerbates Aβ and tau pathologies, potentially accelerating AD progression  [68]. Similarly, peripheral LPS challenge provokes neuroinflammation and DA neuron degeneration, mirroring processes relevant to PD  [69] (Fig. 1). Prolonged exposure to air pollution in children and young adults has been associated with neuroinflammation, altered innate immune responses, and aggregation of Aβ and α-synuclein [70].

Within the context of autoimmune diseases, rheumatoid arthritis, a chronic inflammatory condition with distinct rhythmic immune fluctuations  [71], is associated with a trend of increased risk of AD [72] (Fig. 1). In terms of infection, pathogenic influenza strains, including those responsible for the Spanish Flu pandemic, can elicit marked peripheral immune activation  [73] (Fig. 1). Notably, individuals potentially exposed to highly pathogenic influenza during that flu pandemic at a young age were epidemiologically linked to a higher risk of developing parkinsonism  [74]. Metabolic diseases such as diabetes mellitus and cardiovascular conditions like hypertension, are also tightly coupled to systemic inflammation  [75, 76] (Fig. 1). Midlife hypertension and diabetes, in particular, potentially correlate with higher AD prevalence  [66, 77] (Fig. 1).

Collectively, these findings demonstrate that peripheral inflammatory factors may be associated with increased risk of AD and PD by triggering neuroinflammation, fostering pathological protein aggregation, or disrupting immune homeostasis.

#### The organ-brain axis in modulating neuroinflammation in AD and PD

In addition to systemic influences, specific peripheral organs—most notably the gut and the liver—can convey pro- or anti-inflammatory signals directly to the CNS via anatomical and functional “organ–brain axes”, thereby modulating neuroinflammatory responses (Fig. 1). A nationwide longitudinal study found that inflammatory bowel disease (IBD) is associated with an increased risk of dementia  [78], while the causality in humans remains unproven. Dysbiosis of the gut microbiota compromises the intestinal epithelial barrier integrity and promotes the release of pro-inflammatory cytokines  [79, 80]. In AD, the gut microbiome shows elevated abundance of *Bacteroides*, which potentially drives pro-inflammatory polyunsaturated fatty acid metabolism; this dysbiotic profile contributes to microglial activation in the brain  [79]. Moreover, fecal microbiota transplantation (FMT) from AD-model mice into healthy mice induces neuroinflammation  [81], but substantial research is still needed to explore whether FMT-based strategies can be applied in the treatment of AD and PD [82]. In PD, IBD moderately elevates the disease risk, particularly in individuals aged > 65  [83]. Gut microbiota dysregulation fosters a pro-inflammatory microenvironment that may contribute to PD pathogenesis  [67]. Together, these findings highlight the pivotal role of the gut–brain axis in neuroinflammation in both AD and PD (Fig. 1).

Moreover, liver dysfunction is associated with gut microbiota dysbiosis, involving impaired short-chain fatty acid metabolism and intestinal barrier dysfunction [84]. Intestinal dysbiosis, coupled with compromised barrier integrity, facilitates bacterial translocation and endotoxemia, triggering systemic inflammation that extends into the brain parenchyma and fuels neuroinflammatory cascades [84]. These findings underscore the importance of the liver–gut–brain axis in regulating neuroinflammatory responses (Fig. 1). In parallel, chronic liver inflammation potentially modulates brain immune activity and cognitive performance through multiple mechanisms, including the release of immune-related mediators [85, 86], highlighting the importance of the liver–brain axis in shaping both neuroinflammation and cognitive function.

Notably, peripheral organ–brain axes, such as the gut–brain and the gut–liver–brain axes, potentially modulate CNS inflammation partly by affecting the BBB integrity  [84, 87]. A case in point is chronic liver disease, where gut microbiota dysbiosis and impaired intestinal barrier integrity enhance the translocation of bacteria and microbial metabolites to the liver via the portal circulation. These pathogen-associated molecular patterns engage TLR4 on hepatic reticuloendothelial cells, activating NF-κB and MyD88 (myeloid differentiation primary response 88) signaling cascades. This culminates in the release of pro-inflammatory cytokines, driving systemic inflammation and contributing to BBB dysfunction  [84]. Together, these findings indicate that peripheral organ–brain axes exert a critical influence on neuroinflammation and the pathogenesis of AD and PD, likely involving BBB disruption.

Current evidence demonstrates that peripheral organ–brain axes modulate neuroinflammation and consequently affect the progression of AD and PD. Nevertheless, the precise molecular mechanisms underlying these effects, as well as the interrelationships among distinct organ–brain axes, remain poorly defined. It should be noted that the epidemiological links between systemic/metabolic conditions and AD/PD do not establish causality in humans. Mechanistic insights from animal models require validation in clinical settings. Further research is needed to elucidate how these axes intersect with neuroinflammation driven by AD- and PD-related pathological proteins. A deeper understanding of these interactions will be instrumental in guiding the development of targeted immunomodulatory and neuroprotective therapies.

In addition, FMT from APP/PS1 mice into wild-type mice was sufficient to elicit AD-like cognitive deficits and neuroinflammation  [88]. Emerging evidence further suggests that the gut may represent an early site of pathological protein aggregation in both AD and PD. However, such findings remain model-dependent and have not been validated for human disease initiation. In a gut-inducible transgenic mouse model, α-synuclein or tau pathology initially emerges in enteric neurons, then propagates to medullary centers, such as the dorsal motor nucleus of the vagus and the nucleus of the solitary tract, before extending to broader brain regions. Notably, this gut-origin propagation of α-synuclein and tau can precipitate coexisting AD and PD pathologies  [89]. These findings indicate that neuroinflammation in AD and PD may not originate exclusively within the CNS, but can also arise in peripheral organs, such as the gut, via the translocation of pathological proteins.

### Type 2 immunity

Type 2 immunity, traditionally associated with host defense against allergy and parasitic infections, has emerged as a critical regulator of tissue (including CNS) repair and homeostasis [90]. This arm of immunity encompasses a coordinated network of cellular and molecular components, including innate lymphoid type 2 cells (ILC2s), T helper 2 cells, eosinophils, basophils, mast cells, and alternatively activated macrophages. These elements are orchestrated by signature cytokines such as IL-4, IL-5, IL-9, and IL-13, the upstream “alarmins” IL-33 and IL-25, and thymic stromal lymphopoietin [90, 91].

Within the CNS and its borders, particularly the meninges and choroid plexus, ILC2s act as early responders. They are primarily tissue-resident cells enriched at barrier sites, notably the meninges and the choroid plexus, positioning them at the neuro-immune interface to sense and respond to insults [91, 92]. Activated by alarmins IL-33 and IL-25, ILC2s orchestrate a type 2 immune program. They produce effector cytokines, such as IL-5 and IL-13, as well as amphiregulin, thereby regulating neuroinflammation and contributing to tissue repair [92]. Through these mechanisms, ILC2s have emerged as a pivotal regulator of neuroimmune function, with implications for both aging and neurodegenerative diseases.

Accumulating evidence indicates that ILC2s accumulate in the choroid plexus of aged brains. Transplantation of activated ILC2s into the cerebral ventricles rejuvenated the aged mouse brain and improved cognitive performance. Importantly, administration of IL-5, the primary cytokine produced by ILC2s, significantly suppressed age-related neuroinflammation and mitigated the associated cognitive decline [93]. In postmortem brains of AD patients, alterations in IL-5 levels correlate with pathological severity [94]. IL-33 regulates the innate immune responses by polarizing microglia/macrophages toward an anti-inflammatory phenotype while reducing proinflammatory gene expression, contributing to alleviation of AD-like pathology and cognitive dysfunction [95]. Extending these findings to PD, previous studies have reported that a single nucleotide polymorphism in IL-13 from individuals with idiopathic PD increases cellular susceptibility to oxidative stress [96]. Given that IL-33 promotes microglial phagocytosis and alternative activation [95], it is plausible that enhancing type 2 immune responses may confer benefits in PD. Nevertheless, direct evidence for type 2 immune dysregulation in PD remains limited. Consequently, strategies aimed at enhancing type 2 immunity, such as IL-33 supplementation and ILC2 transfer, are emerging as promising therapeutic approaches to restore neuroimmune homeostasis and counteract neurodegeneration.

## Sensors in the brain

### Microglia

**Fig. 2:**
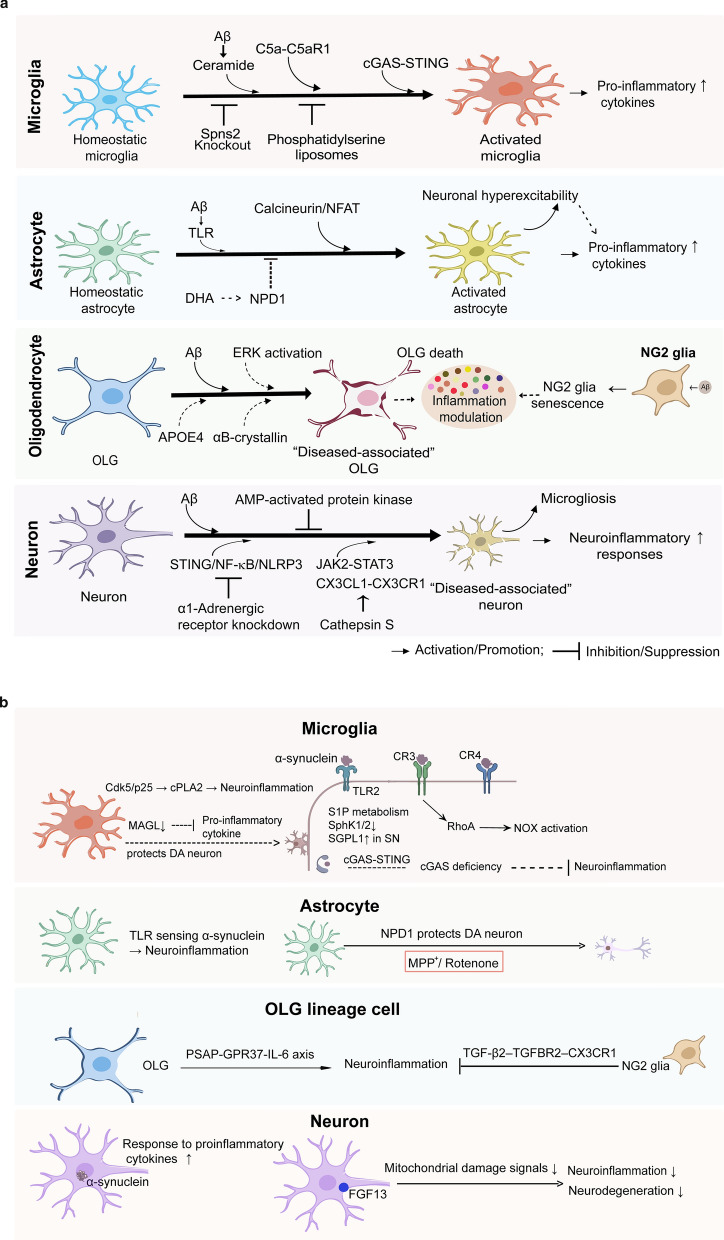
Cellular sensors and potential related pathways underlying neuroinflammation in AD and PD. Microglia, astrocytes, oligodendrocyte lineage cells, and neurons act as cellular sensors that mediate neuroinflammation in AD (**a**) and PD (**b**). **a** Microglia are associated with lipid metabolism, complement system, and cGAS-STING signaling. Astrocytes are potentially related to calcineurin/nuclear factor of activated T cells (NFAT), TLR cascades, and lipid mediators including DHA/NPD1. Oligodendrocyte lineage cells and neurons may be involved in glial inflammatory networks and neuroinflammatory regulation via multiple signaling pathways. **b** Microglia are indicated to be linked to lipid metabolism, complement system, and cGAS-STING signaling. Astrocytes are related to TLR cascades and NPD1‑mediated inflammatory regulation. Oligodendrocyte lineage cells are involved in the PSAP-GPR37-IL6 axis, and NG2 glia show anti‑inflammatory and neuroprotective profiles. Neurons are associated with α‑synuclein‑related responses and neuroinflammatory modulation. PSAP: prosaposin

Microglia are resident macrophages in the CNS and play an indispensable role in maintaining homeostasis and orchestrating immune defense. Under physiological conditions, they adopt a ramified, “resting” morphology and remain in a constant surveillance mode, continuously monitoring the local microenvironment. In response to injury or pathological stimuli, microglia rapidly shift to an activated, amoeboid state  [97] (Fig. 2a). The activated microglia have been categorized into two opposing phenotypes: pro-inflammatory (M1) and anti-inflammatory (M2), based on their distinct cytokine expression patterns [98]. The M1 microglia contribute to neuroinflammatory responses, while the M2 subtype is primarily involved in the phagocytosis of misfolded proteins, maintenance of neuronal survival, and promotion of tissue repair [99].

Recent studies, however, have challenged this binary classification, viewing microglia as a highly heterogeneous population with diverse intrinsic properties and functional specializations  [100–102]. In AD, scRNA-seq has revealed a novel microglial subtype, termed disease-associated microglia (DAM), characterized by downregulation of homeostatic genes (e.g., *P2ry12*, *Cx3cr1*) and upregulation of genes involved in phagocytosis and lipid metabolism (e.g., *Apoe*, *Lpl*, *Trem2*). These cells localize near Aβ plaques, exhibit phagocytic activity by engulfing amyloid particles, and are suggested to play a protective role in restricting neurodegeneration [60, 103]. Subsequent research shows that DAM arises from excessive microglial proliferation, which drives these cells into replicative senescence. These senescent DAM display telomere shortening, increase of senescence-associated β-galactosidase activity, and a transcriptional signature associated with senescence. Preventing early microglial proliferation blocks both senescence and DAM formation, leading to reduce Aβ pathology. Thus, while DAM possess phagocytic capacity, their senescent phenotype may contribute to early amyloid pathology, revealing a complex, stage-dependent role in AD [104]. Spatially restricted to neuritic Aβ plaques and mechanistically driven by the TREM2-APOE pathway, homeostatic microglia lose their identity and transform into a neurodegenerative phenotype (MGnD) characterized by downregulation of P2ry12/TMEM119 and upregulation of APOE and Clec7a [103, 105]. In addition to DAM and MGnD, a distinct population of TREM2-expressing senescent microglia has been identified in aging and AD models. Eliminating these cells improved cognitive function and reduced neuroinflammation [106]. Moreover, single-cell analysis of human AD brains has revealed a rich diversity of microglial states beyond the homeostatic phenotype, including lipid-processing (MG4), phagocytic (MG5), and transcriptionally distinct inflammatory states (MG2, MG8, MG10) that exhibit dynamic changes across disease progression [107], illustrating the complex roles of microglia in neurodegeneration. In addition, in vivo α-synuclein overexpression induces DAM, indicating the potential roles of DAM in PD [108]. The existence and functions of other microglial phenotypes in PD warrant further investigation.

Beyond the classical receptor-mediated activation cascades, emerging evidence implicates lipid metabolism pathways as critical drivers of microglial neuroinflammation associated with AD and PD (Fig. 2a and b), such as cytosolic phospholipase A2 (cPLA2, encoded by *PLA2G4A*) and monoacylglycerol lipase (MAGL) signaling [109–113]. cPLA2 catalyzes the hydrolysis of membrane phospholipids at the sn-2-position to release arachidonic acid [114, 115], which is subsequently metabolized into a diverse family of eicosanoid lipid mediators (e.g. prostaglandins, leukotrienes and epoxyeicosatrienoic acid) that are involved in neuroinflammation [109, 116]. Upregulation of cPLA2 promotes neuroinflammation across multiple neurodegenerative models [117], including AD models. Additionally, specific ablation of the peroxisomal fatty acid β-oxidation enzyme MFE-2 in microglia leads to lipid accumulation, resulting in excessive production of arachidonic acid, mitochondrial reactive oxygen species, and pro-inflammatory cytokines [118]. In AD models, pharmacological inhibition of MAGL suppresses Aβ production and reduces neuroinflammation [112, 113]. In PD models, Cdk5/p25 hyperactivation promotes cPLA2 upregulation, leading to lysophosphatidylcholine (LPC) production, thereby contributing to neuroinflammation [117] (Fig. 2b). Inhibition of MAGL reduces the expression of prostaglandin and pro-inflammatory cytokines, and protects DA neurons from 1-methyl-4-phenyl-1,2,3,6-tetrahydropyridine (MPTP)-induced neurotoxicity [119] (Fig. 2b). In addition, mutations in *GBA1*, the most significant genetic risk factor for PD, disrupt sphingolipid metabolism and lysosomal function, promoting pro-inflammatory cytokine production in microglia and enhancing astrocytic activation, thereby exacerbating α-synuclein pathology [120, 121].

Emerging evidence highlights ceramide, a key sphingolipid mediator, as a central contributor to microglial-associated neuroinflammation. In AD models, Aβ induces ceramide generation in microglia through acid sphingomyelinase (ASM) activation [122, 123]. This ceramide production promotes the release of pro-inflammatory cytokines (e.g., C1q, TNF-α, IL-1α) from microglia (Fig. 2a), which subsequently influence astrocytic reactivity (discussed below) [123]. Consistent with this, pharmacological inhibition of ASM in 5 × FAD mice reduces microglial cytokine secretion and mitigates AD-related pathology [123]. Another key effector of the sphingolipid pathway is sphingosine-1-phosphate (S1P), which is generated from ceramide via the sequential actions of ceramidase and sphingosine kinases (Sphk1 and Sphk2). The levels of S1P are tightly regulated by the balance between Sphk1/2 and S1P lyase (Sgpl1) [124, 125]. S1P plays a key role in modulating microglial migration, activation, and survival through its receptors, particularly S1pr1 (also known as S1P1) [110]. In AD models, the S1P transporter Spns2 promotes pro-inflammatory microglial activation in response to Aβ42. Spns2 knockout reduces the Aβ42-induced NF-κB activation and pro-inflammatory cytokine production (Fig. 2a), thereby improving working memory in Aβ42-injected mice [126]. The S1pr1 antagonist Ponesimod reduces the TLR4-induced neuroinflammation and enhances Aβ clearance in 5 × FAD mice [127]. In PD, downregulation of SphK1 and SphK2, and upregulation of SGPL1, have been observed in the SN (Fig. 2b), suggesting that altered S1P metabolism may contribute to microglial-mediated neuroinflammation in PD pathology [128].

Moreover, phosphoinositides, the phosphorylated derivatives of phosphatidylinositol, regulate microglia-mediated neuroinflammation and neurodegeneration, with disrupted phosphoinositide homeostasis implicated in AD and PD [111]. Furthermore, beyond endogenous lipid metabolism, exogenous phosphatidylserine-containing liposomes have been shown to inhibit the Aβ- and IFN-γ-induced microglial activation and pro-inflammatory cytokine production [129] (Fig. 2a). Collectively, these findings indicate that multiple lipid-related pathways, including eicosanoid, ceramide, S1P, phosphoinositides, and exogenous phosphatidylserine, converge on microglial activation and neuroinflammation across both neurodegenerative diseases.

During AD progression, key homeostatic functions of microglia, such as phagocytosis of Aβ aggregates and synaptic elimination, become dysregulated  [130, 131]. Advanced pathology is frequently accompanied by region-specific alterations in microglial distribution, morphology, and functionality. Nevertheless, the spatiotemporal dynamics of these phenotypic transitions in neurodegenerative diseases such as AD and PD remain incompletely understood.

Clinical evidence underscores the microglia-mediated neuroinflammation as a defining feature of both AD and PD  [132, 133]. Microglia express a broad repertoire of pattern recognition receptors, including TLRs, nucleotide-binding oligomerization domain (NOD)-like receptors, and receptors for advanced glycation end products (RAGE), which potentially detect danger-associated molecular patterns, such as Aβ  [134, 135]. Engagement of TLR4 stimulates pro-inflammatory cytokine production and regulates Aβ uptake [136, 137], whereas Aβ activates the NLRP3 inflammasome to amplify inflammatory signaling  [138]. In addition, Aβ interaction with RAGE induces microglial activation  [139, 140]. Notably, receptors, such as CD14 and mFPR2, also sense Aβ and regulate its internalization [141, 142].

The complement system, a central pillar of the innate immunity, is closely intertwined with neuroinflammation in AD. Complement proteins, such as C1q and C3, are spatially colocalized with Aβ plaques in both AD patients and experimental models. Alterations in the expression of complement components, including C1q and C3, have been documented in AD [143, 144]. Pathological forms of Aβ and tau activate complement cascades  [143]. C1q, in particular, participates in microglial neuroinflammatory responses  [145, 146].

Synaptic accumulation of Aβ or tau upregulates C1q in neighboring microglia, triggering complement activation at synapses and promoting subsequent phagocytic removal  [130]. Prevention of the C5a–C5aR1 pathway diminishes microglial and astrocytic activation, and improves cognitive performance in AD mouse models  [1, 147, 148] (Fig. 2a). Together, these findings identify complement components, such as C1q, C3, and the C5a–C5aR1 axis, as key mediators of Aβ/tau-associated neuroinflammation (Fig. 2a), highlighting the importance of defining their context-dependent functions to develop targeted therapies that preserve homeostatic immune responses.

Similar to AD, complement components, such as C3, exhibit altered expression in PD  [143, 144]. α-Synuclein potentially modulates microglial inflammatory responses through engagement of multiple receptors including TLR2  [149] (Fig. 2b), as well as complement receptors CR3 (CD11b/CD18 integrin) and CR4 (CD11c/CD18 integrin), leading to cytokine release or enhanced microglial phagocytosis  [144] (Fig. 2b). The α-synuclein–CD11b interaction regulates microglial NADPH oxidase (NOX2) activation through RhoA pathways  [144, 150] (Fig. 2b). Genetic deletion of CD11b attenuates the α-synuclein-induced NOX2 activation in microglia [150]. These findings collectively demonstrate that α-synuclein participates in microglial inflammation in PD by regulating microglial activation and migration, and NOX-related signaling cascades, with CD11b serving as a pivotal modulator. Elucidating how α-synuclein conformation influences these receptors may uncover novel therapeutic entry points for attenuating neuroinflammation while preserving microglial homeostatic functions.

Beyond TLRs and complement receptors, α-synuclein engages additional microglial receptors, such as prostaglandin E receptor subtype 2 and CD36, to modulate inflammatory activation  [151]. Microglia also express a wide array of purinergic receptors that detect damage‑associated signals released from injured or dying neurons, thereby promoting inflammatory signaling  [152, 153]. For example, inhibition of the P2Y12 receptor markedly reduces LPS-induced expression of pro-inflammatory factors  [154].

The cyclic GMP-AMP synthase (cGAS)–stimulator of interferon genes (STING) pathway is a core component of the innate immune system that detects double-stranded DNA from both invading pathogens and damaged host cells  [155, 156]. Upon binding to double-stranded DNA, cGAS catalyzes the synthesis of the second messenger cyclic GMP-AMP, which binds and activates STING localized on the endoplasmic reticulum. This triggers STING oligomerization and its trafficking to the Golgi apparatus, where it recruits TBK1 and IRF3 to induce type I interferon production, while also engaging NF-κB signaling and non-canonical autophagic pathways  [156]. Dysregulation of this cascade is implicated in a wide range of diseases, including infections, autoimmune and inflammatory disorders, cancer, and neurodegenerative conditions  [157, 158].

Mounting evidence has now linked cGAS–STING signaling to the pathogenesis of AD and PD. In AD, cGAS and STING are markedly upregulated in microglia clustering around Aβ plaques [159], and activation of the cGAS–STING axis has been documented in AD brains  [160]. Genetic deletion of microglial cGAS significantly reduces Aβ-related pathology [161], while certain AD risk variants exacerbate the cGAS-driven microglial senescence and neurodegeneration in tauopathy models  [162]. In PD, the MPTP-induced mouse model exhibits robust cGAS–STING pathway activation, and loss of cGAS mitigates MPTP neurotoxicity [163]. Additionally, cGAS deficiency inhibits the expression of antiviral-related inflammatory genes in MPTP-treated microglia  [163] (Fig. 2b). Another line of evidence shows that mitophagy protects against the manganese-induced parkinsonism by dampening microglial neuroinflammation through the mitochondrial DNA–STING axis [164].

Notably, cGAS expression is higher in microglia compared to other neural cell types, including neurons, oligodendrocyte precursor cells (OPCs), and astrocytes  [163, 165], highlighting the specialized role of microglial cGAS–STING signaling in these neurodegenerative contexts. Moreover, pharmacological inhibition of the cGAS–STING pathway blunts the LPS-induced M1 microglial polarization [166]. Collectively, these findings indicate that the cGAS–STING pathway is involved in regulating AD and PD pathology largely by shaping microglial neuroinflammatory responses (Fig. 2a, b).

In summary, microglia detect diverse pathological cues, including Aβ, tau, and α-synuclein, through an intricate receptor network, driving neuroinflammation in AD and PD. Nevertheless, fundamental questions persist. How are the spatial and temporal features of microglial subtypes or activation states determined? How are diverse microglial receptor signaling pathways integrated to shape the phenotypic outcomes during neurodegeneration? Dissecting these complex and finely balanced processes is essential for avoiding off-target immune perturbation in therapeutic strategies for AD and PD.

### Astrocytes

Astrocytes account for approximately 40% of all brain cells in humans [167]. They function as essential regulators of neural homeostasis far beyond their classical roles in providing metabolic and nutritional support for neurons  [168]. Growing evidence has unveiled their multifaceted roles in the modulation of synapse formation, synaptic plasticity, and cognitive processes, highlighting their integral role in overall brain function  [168, 169].

Astrocytes display considerable morphological and regional heterogeneity and are broadly classified into two principal subtypes: fibrous astrocytes and protoplasmic astrocytes. Fibrous astrocytes are primarily located in white matter, where they support axonal myelination and maintain structural integrity. In contrast, protoplasmic astrocytes are densely distributed in gray matter and extend perisynaptic processes that ensheathe synapses, thereby regulating synaptic development. Additionally, through their endfeet contacts with blood vessels, astrocytes help preserve BBB integrity. Together, these specialized functions position astrocytes as a key integrator of neural and vascular signaling networks  [170, 171].

Under pathological conditions, such as injury or infection, astrocytes become activated, characterized by pronounced changes in morphology, molecular expression, and functional output. Similar as microglial polarization, a recent statement has challenged the classification of reactivated astrocytes as A1 (neurotoxic) and A2 (neurotrophic) phenotypes [172, 173], arguing that this binary model is overly reductive and fails to reflect the dynamic phenotypic plasticity of glial cells under pathological conditions [20].

Accumulating evidence positions astrocytes as active contributors to the inflammatory processes in AD and PD  [173]. Reactive astrogliosis has been documented in the brains of patients with both disorders  [174, 175]: in AD, astrocytes cluster around Aβ aggregates, whereas in PD they encircle degenerating neurons  [174, 176]. Pathological Aβ influences astrocyte biology through multiple mechanisms. For example, oligomeric and fibrillar Aβ promotes APP processing via β-secretase in primary cultured astrocytes  [177]. In addition, existing research implies that Aβ may trigger neuronal toxicity through a cascade involving elevated intracellular calcium, mitochondrial dysfunction, and glutamate release within astrocytes. [178–180].

Conversely, activated astrocytes can mitigate Aβ burden by phagocytosis [181]. Astrocytic calcineurin/NFAT (nuclear factor of activated T cells) signaling induces neuronal hyperexcitability in AD mouse models  [182] and may also contribute to inflammatory responses  [183] (Fig. 2a). Similar to microglia, astrocytes sense Aβ and α-synuclein via TLR pathways, inducing neuroinflammatory cascades  [184, 185] (Fig. 2a and b). The ε4 allele of apolipoprotein E (*APOE4*), the strongest genetic risk factor for late-onset AD, enhances Aβ aggregation, and APOE isoforms differentially regulate astrogliosis and neuroinflammation in AD  [186]. Additionally, multiple intracellular signaling cascades, including JAK-STAT3, MAPK, and NF-κB, mediate astrocyte-associated neuroinflammation in AD  [187]. Aβ-induced inflammatory signaling is also linked to MAPK activation  [24]. Collectively, these findings highlight the central role of aforementioned signaling pathways in regulating Aβ/tau-associated neuroinflammation within astrocytes.

Beyond classical signaling pathways, emerging evidence points to ceramide as a key modulator of astrocytic reactivity through lipid metabolic dysregulation [188]. Under neuroinflammatory stress, ceramides accumulate in astrocytes [189]. In the 5 × FAD mouse model of AD, reactive astrocytes exhibit increased ceramide generation, and inhibition of ASM reduces the secretion of ceramide-enriched extracellular vesicles from these cells [123]. These astrocyte-derived extracellular vesicles induce neuronal toxicity and impair mitochondrial respiration capacity in neurons. Pharmacological inhibition of ASM in 5 × FAD mice reduces the secretion of these mitotoxic extracellular vesicles and ameliorates AD-related pathology [123]. In PD dementia, post-mortem analysis revealed increased ceramide levels in astrocytes and a shift toward pro-apoptotic ceramide species, indicating the potential role of astrocyte-derived ceramide in regulating neuroinflammation [188]. Consistent with the ceramide-mediated microglial activation discussed earlier, astrocytes also exhibit ceramide-dependent dysregulation, further amplifying neuroinflammatory cascades [123].

In contrast to the pro-inflammatory effects of ceramide, astrocytes also produce docosahexaenoic acid (DHA, 22:6n-3), an omega-3 polyunsaturated fatty acid that actively counters neuroinflammation. Although the brain obtains most of its DHA from the diet and liver, astrocytes (along with cerebral endothelial cells) are capable of synthesizing DHA locally [190–192]. Within astrocytes, DHA is metabolized into specialized pro-resolving mediators, including neuroprotectin D1 (NPD1), which actively resolves neuroinflammation. In AD, NPD1 levels are reduced in the brains of patients, particularly in the hippocampus [193, 194]. NPD1 deficiency has also been implicated in PD pathogenesis. NPD1 has been shown to protect DA neurons from MPP⁺- or rotenone-induced apoptosis in a cellular model [194, 195] (Fig. 2b). Mechanistically, DHA suppresses astrocytic pro-inflammatory responses by inhibiting NF-κB and AP-1 activation, thereby reducing the production of TNF-α and IL-6 [196] (Fig. 2a). Thus, astrocytic DHA metabolism provides a neuroprotective counterbalance to ceramide-driven neuroinflammation in both AD and PD.

In summary, astrocytes are closely linked to the neuroinflammation landscape of AD and PD, capable of detecting pathological stimuli such as Aβ and α-synuclein. Upon activation, they undergo phenotypic switching and regulate inflammatory responses through key signaling cascades, emphasizing their central position in disease-related immune dynamics. Nevertheless, the intricate crosstalk between microglial and astrocytic subtypes during neuroinflammation remains poorly understood; deciphering these interactions may shed light on disease pathogenesis and development of targeted therapies aimed at modulating astrocytic function to alleviate neuroinflammation. Furthermore, clarifying the functional distinctions and context-dependent activities of A1 versus A2 astrocytes may enable selective induction of neuroprotective phenotypes, opening novel avenues for therapeutic intervention.

### Oligodendrocyte (OLG) lineage cells

OLG lineage cells consist of two principal populations: OLGs and mature OPCs, the latter also referred to as NG2 glia owing to their expression of the NG2 chondroitin sulfate proteoglycan  [197]. The NG2 glia represent the principal progenitor reservoir for OLGs and maintain the ability to proliferate and differentiate into OLGs under both physiological settings, such as developmental myelination, and pathological conditions including remyelination following demyelination  [198].

Beyond the progenitor role, NG2 glia form functional synaptic contacts with neurons, influencing neuronal plasticity as well as their own proliferation and differentiation. Therefore, NG2 glia actively participate in neural circuit homeostasis  [199, 200]. In contrast, mature OLGs are primarily responsible for producing myelin sheaths that wrap axons, a process indispensable for salutatory conduction and axonal preservation  [197]. Growing evidence positions both NG2 glia and OLGs as key contributors to the pathogenesis of neurological diseases  [197, 201]. Recent investigations have challenged the traditional view of OLGs by demonstrating their substantial involvement in disease-associated neuroinflammation  [202, 203].

Traditionally viewed as dedicated myelinating cells, OLGs are now recognized as active participants in neuroinflammatory processes. Dysregulation of APP, whether through deficiency or overexpression, alters myelin sheath thickness, directly coupling APP metabolism to OLG function  [204]. Exposure to Aβ aggregates induces OLG death  [205] (Fig. 2a). In Aβ-rich environments, the expression of myelination-related genes follows a biphasic pattern: an initial upregulation followed by sustained downregulation  [206]. White matter disruption is detectable already in preclinical AD  [207], suggesting that OLG dysfunction is an early pathogenic event. However, the underlying molecular mechanisms remain to be fully elucidated.

Studies have shown that protein disulfide isomerase, a key regulator of inflammation, and its isoform P5, are markedly reduced in OLGs of AD brains  [208, 209]. Single-nucleus transcriptomic analyses have uncovered differentially expressed genes in AD-associated OLGs  [61, 210]. scRNA-seq has identified a distinct OLG subpopulation in AD characterized by extracellular signal-regulated kinase (ERK) signaling–mediated activation  [211]. In parallel, chromatin and transcriptomic profiling has linked OLG-associated cis-regulatory elements to AD risk loci such as *APOE* and *CLU*  [212]. The *APOE4* allele disrupts myelination by impairing cholesterol homeostasis in OLGs  [213] (Fig. 2a).

Given that both ERK signaling and APOE are implicated in neuroinflammatory processes  [214–216], these findings suggest that OLGs may modulate AD-associated inflammation through ERK- and APOE-dependent pathways (Fig. 2a). Moreover, single-cell transcriptomic studies have demonstrated elevated expression of αB-crystallin in OLGs from individuals with AD  [217]. αB-crystallin regulates astrocytic dopamine D2 receptor-mediated neuroinflammation and microglial cytokine secretion  [218, 219], implicating that OLGs may participate in the coordination of glial inflammatory networks via αB-crystallin signaling (Fig. 2a). Additionally, OLGs can sense metabolic stress, such as glycolytic impairment, which may trigger inflammasome activation in the AD context  [220]. Collectively, these results indicate that OLGs respond to AD pathology in part by engaging in neuroinflammatory regulation.

In PD, OLGs are likewise implicated, as their numbers are reduced in the midbrain of affected patients  [202]. Human single-nucleus transcriptomic analyses have linked PD risk to OLG-specific gene expression signatures  [221]. Notably, a recent study demonstrated that OLGs can contribute to neuroinflammatory cascades that may exacerbate neurodegeneration in PD models (Fig. 2b), representing a paradigm shift in our understanding of their role beyond classical myelination  [13].

Nevertheless, key questions remain: How do OLGs respond to α-synuclein inclusions? Do their inflammatory functions in PD overlap with or diverge from those in AD? Earlier work has shown that nigral DA neurons possess long, unmyelinated axons  [222]; thus, OLG dysfunction observed in PD may reflect a non-cell-autonomous response to alterations in the local microenvironment rather than a direct consequence of DA neuron pathology. These findings suggest that changes in the SN milieu contribute to PD pathogenesis.

NG2 glia are increasingly appreciated as versatile sentinels of CNS disturbances, mounting dynamic responses to a range of insults, including PD-associated neurodegeneration  [223], mechanical injury such as stab wounds, and ischemic stroke  [224, 225]. Clinical and animal studies have revealed that NG2 glia adopt a senescence phenotype in regions within and surrounding Aβ plaques. In culture, Aβ aggregates directly induce senescence in NG2 glial cells  [226] (Fig. 2a), indicating that these progenitors are primary responders to Aβ-mediated stress. Parallel work has described an “activated” NG2 glial phenotype, typified by enlarged cell bodies, as well as retracted and less branched processes, implying context-dependent functional plasticity  [227].

In a rat model of PD, NG2 glia display an activated phenotype in the SN  [228]. Furthermore, in levodopa-induced dyskinesia, the NG2 glia population was reduced in the lesioned striatum, and the surviving cells adopted a reactive phenotype, implicating the potential functions of these progenitors in treatment-related neuroplasticity  [229]. Notably, our previous work showed that NG2 glia suppress microglial activation through the TGF-β2 (transforming growth factor-β2)–TGFBR2 (TGF-β type II receptor)–CX3CR1 (CX3C chemokine receptor 1) signaling (Fig. 2b). Furthermore, genetic deletion of NG2 glia exacerbated neuroinflammation in LPS-treated mice, supporting their anti-inflammatory function. NG2 glia deficiency accelerated DA neurons degeneration in the SN of PD mice, highlighting their neuroprotective potential [223]. Although these findings demonstrate that NG2 glia contribute to anti-inflammatory processes, several questions remain unresolved, including whether distinct functional subtypes of NG2 glia exist, and what is the molecular basis of their crosstalk with microglia or astrocytes during neuroinflammatory cascades.

### Neurons

Neurons are not merely passive victims in the neuroinflammatory cascades underlying AD and PD. Rather, they are active participants that both instigate and respond to pathological alterations. In AD, fibrillar Aβ increases cytokine secretion in differentiating neurospheres [230]. Evidence indicates that intraneuronal accumulation of Aβ triggers a neuron-derived inflammatory response prior to extracellular plaque formation and cell death. Moreover, neurons burdened with Aβ upregulate proinflammatory and chemotactic factors (Fig. 2a), which potentially correlate with microglial recruitment and activation. Neuronal AMP-activated protein kinase inhibits lipogenesis and promotes lipophagy in neurons, thereby reducing lipid flux to microglia and ameliorating microgliosis [231] (Fig. 2a). These findings suggest that neurons themselves may act as a primary responder of neuroinflammation in early AD, distinct from plaque-associated glial responses [232]. In PD, α-synuclein potentially enhances neuronal response to proinflammatory cytokines [233] (Fig. 2b). Furthermore, neuron-like membrane-coated nanoparticles fabricated via microfluidics from mesenchymal stem cells have been shown to suppress inflammation and apoptosis in PD [234].

Additionally, neuron-associated pathways contribute to the modulation of neuroinflammation. In AD, neuronal knockdown of α1-adrenergic receptor attenuates neuroinflammation and tauopathy by suppressing the STING/NF-κB/NLRP3 axis [235] (Fig. 2a). Neuronal cathepsin S promotes neuroinflammatory responses and contributes to cognitive impairment via the CX3CL1–CX3CR1 axis and JAK2–STAT3 signaling [12] (Fig. 2a). In PD, neuronal FGF13 (fibroblast growth factor 13) limits neuroinflammation and prevents neurodegeneration by inhibiting mitochondria-derived damage signals [236] (Fig. 2b). Collectively, these results reveal that neuronal signaling pathways play important roles in mediating AD- and PD-associated neuroinflammation.

Notably, neuronal lipid composition also directly shapes the neuroinflammatory environment. Beyond their structural roles, specific phospholipids actively participate in inflammatory signaling. For instance, phosphatidylserine binds to various proteins and is involved in neuroinflammation, neurotransmission, and synaptic refinement [237]. Phosphatidylinositol and its phosphorylated derivatives are also key players in neuroinflammatory cascades, acting as precursors to second messengers and regulators of membrane signaling [111]. In AD patients, the fatty acid composition of neuronal membrane phospholipids, particularly phosphatidylserine and phosphatidylinositol, shows significant alterations in the parahippocampal cortex [238]. In PD, induced pluripotent stem cell (iPSC)-derived DA neurons carrying *MAPT* mutations exhibit significantly higher levels of oxidized phospholipids compared to WT neurons [239]. Such lipid compositional changes in both AD and PD may influence neuronal signaling and glial responses, although the precise mechanisms remain to be elucidated [238, 239].

### Crosstalk between neural cells

Under physiological conditions, bidirectional communication between neurons and glial cells constitutes a highly orchestrated network essential for maintaining brain homeostasis and supporting functions from synaptic plasticity to immune quiescence  [240, 241]. For example, astrocytes supply glutamine to GABAergic neurons via the glutamine–glutamate cycle, illustrating the metabolic interdependence that preserves neurotransmitter balance  [242]. At the synaptic level, hippocampal long-term potentiation induces dynamic remodeling of microglial processes and prolongs their contact with dendritic spines, indicating that microglia actively participate in synaptic plasticity  [243]. These interactions are regulated by diverse ligand-receptor signaling pathways  [240]. Microglia-derived IL-1β, acting through neuronal IL-1 receptor, modulates neuronal activity by enhancing glutamate release  [240, 244]. Engagement of microglial CD200R by neuronal CD200 constrains excessive microglial activation, thereby preserving CNS immune homeostasis [241, 245]. Neuronal C3 mediates synaptic pruning via interaction with microglial C3R  [241, 246]. Glia–glia crosstalk further integrates these networks: microglial TNF-α promotes glutamate release from astrocytes, linking glial activation to potential neuronal excitotoxicity [247]. Together, these findings reveal that intricate neuron–glia and glia–glia interactions constitute a fundamental framework for brain homeostasis. Disruption of these finely tuned circuits impairs neural function and offers critical insights into the pathogenesis of neurodegenerative diseases.

Accumulating evidence indicates that dysregulated neuroinflammation, linked to aberrant crosstalk among neuronal and glial cells, contributes to the pathogenesis of AD and PD  [248, 249]. Over a decade ago, a model was proposed in which microglia serve as primary responders to diverse pathological stimuli, including LPS, Aβ, and α-synuclein  [153, 250]. This activation triggers a pro-inflammatory cascade: activated microglia release cytokines that secondarily activate astrocytes, thereby amplifying the production of inflammatory mediators and ultimately promoting neurodegeneration  [153, 250]. Subsequent studies have corroborated this model by demonstrating that activated microglia can induce the formation of neurotoxic, A1-type reactive astrocytes  [19]. Beyond this feed-forward inflammatory loop, microglia also contribute to AD pathology by mediating tau propagation via phagocytic uptake and exocytic release at synaptic clefts [248], directly linking neuroinflammation to tauopathy progression.

Key signaling pathways governing the neuron–glia crosstalk are frequently disrupted in AD and PD, intensifying neuroinflammatory responses. Among these, the CD200–CD200R and CX3CL1–CX3CR1 axes are well-established regulators of brain immune homeostasis [245, 251]. The CD200–CD200R axis functions as a critical brake on microglial activation. Its expression is reduced in AD brains and in the midbrain of PD mice  [252, 253], and disruption of this pathway enhances microglial reactivity and promotes DA neuron loss in PD  [254]. Conversely, activation of the CD200–CD200R signaling markedly attenuates inflammation induced by both Aβ and α-synuclein  [253, 255]. The CX3CL1–CX3CR1 pathway represents another vital conduit for neuron–microglia communication and exhibits stage-specific dysregulation in AD.

CX3CL1 is elevated in injured neurons within AD brains, and plasma CX3CL1 levels are higher in AD patients with mild-to-moderate symptoms than in those with severe disease, suggesting a link to disease progression  [256]. Genetic ablation of this pathway exacerbates tau phosphorylation, microglial activation, and cytokine overproduction in AD models  [257]. In PD, subthalamic nucleus deep-brain stimulation mitigates neuroinflammation by modulating the CX3CL1/CX3CR1 signaling  [258], highlighting the conserved immunoregulatory role of this pathway across neurodegenerative contexts.

Emerging evidence also implicates OLG lineage cells in the neuron–glia regulatory networks. In PD, elevated prosaposin (PSAP) in the CSF binds to GPR37 on OLGs to induce IL-6 release, thereby initiating neuroinflammation  [13] (Fig. 2b). Notably, IL-6 produced by OLGs can stimulate microglia to release additional IL-6, amplifying the inflammatory cascade and accelerating DA neuron degeneration  [13]. This work uncovers a critical DA neuron–OLG interaction that modulates neuroinflammation via the PSAP–GPR37–IL-6 axis (Fig. 2b). In parallel, NG2 glia exhibit anti-inflammatory properties by restraining microglial activation (Fig. 2b), as microglia exhibit a heightened state of activation in the NG2 glia-ablated brain following LPS treatment  [223]. Together, these findings suggest that in the early stages of AD and PD, an imbalance within the OLG-NG2 glia –microglia axis disrupts brain immune homeostasis. This dysregulation favors dominant pro-inflammatory responses, driven initially by OLGs and subsequently amplified by microglia and astrocytes.

Collectively, neuroinflammation in AD and PD stems not merely from isolated cellular dysfunction, but from a breakdown in dynamic intercellular networks encompassing neurons and glial cells. The inherent complexity of these networks implies that targeting a single cell type or pathway may provide only limited therapeutic efficacy. Future research should aim to delineate how these interactions evolve across disease stages and to identify pivotal checkpoints at which pro-inflammatory cascades can be rebalanced, thereby enabling a more nuanced and effective approach to arresting neurodegeneration in AD and PD.

## Peripheral immune cells

Beyond glial cells and neurons, peripheral immune cells, including lymphocytes, monocytes, natural killer (NK) cells, and neutrophils, can infiltrate into the brain parenchyma, where they regulate the progression of neuroinflammation in both AD and PD [259, 260].

T cells are a key element in modulating neuroinflammation [261]. In PD, both CD4^+^ and CD8^+^ T cells infiltrate the SN, with CD8^+^ T cells appearing during prodromal stages (incidental Lewy body disease) and CD4^+^ T cells contributing to neurodegeneration [262, 263]. In AD, T cell infiltration into the brain parenchyma is also evident, though the temporal dynamics are less well defined; both CD4^+^ and CD8^+^ T cells have been detected in association with amyloid pathology [1, 264, 265]. Mechanistically, CD4^+^ T cells recognize antigens (such as α-synuclein) presented by microglial MHC-II, releasing IFN-γ, TNF-α, and IL-1β, leading to neurotoxic microglial responses [266], while CD8^+^ T cells engage neuronal MHC-I molecules to mediate direct cytotoxicity [267]. Th17 cells represent a particularly pathogenic subset in both diseases, infiltrating via LFA-1/ICAM-1 interactions and directly inducing neuronal death [268, 269]. Additionally, regulatory T cells (Tregs), another important cell type of T cells, play a protective role in both disorders [270]. In mouse models of AD, ex vivo expansion and administration of Aβ-specific human Tregs reduce microglial proinflammatory activity and alleviate amyloid pathology [271]. In PD, Tregs reduce DA neuron loss through suppression of microglial responses to stimuli, including aggregated, nitrated α-synuclein in mouse models [272].

Monocyte recruitment also contributes to neuroinflammation in both diseases. In AD, monocytes infiltrate the brain, where the patrolling monocytes internalize Aβ from vascular walls before re-entering the circulation [259, 273, 274]. Similarly, infiltration of CCR2^+^ monocytes into the SN is required for α-synuclein-induced neuroinflammation and neurodegeneration in PD animal models [275, 276]. Soluble CD163, a monocyte-specific marker, is elevated in the CSF and correlates with cognitive decline, reflecting ongoing neuroinflammation in PD [277]. Interestingly, CCR2 deficiency exerts opposing effects in AD and PD, exacerbating amyloid pathology and cognitive deficits in AD [278, 279], while attenuating the α-synuclein-induced neurodegeneration in PD [276]. This suggests that the CCR2-mediated monocyte function may shape neuroinflammatory responses in fundamentally different ways depending on the underlying proteinopathy.

Additionally, NK cells and neutrophils are potentially involved in neuroinflammation in AD and PD. In AD, NK cell function is impaired. Transcriptomic analyses revealed a contraction of the cell compartment and upregulation of senescence-related genes [280–282]. In PD, NK cells internalize and degrade α-synuclein aggregates via the endosomal/lysosomal pathway, and their depletion exacerbates α-synuclein pathology, neuroinflammation, and motor deficits [283–285]. Aging impacts the NK cell function in both diseases, with immunosenescence leading to reduced IFN-γ production and altered subset distribution [282, 285, 286].

Neutrophils adhere to brain capillaries and reduce cortical blood flow in AD [287]. Infiltrating neutrophils release mitochondria and mitochondrial DNA, activating the mitochondrial DNA–STING–NLRP3/IL-1β axis that drives neuroinflammation and neuronal apoptosis [288]. A recent study identified a new subset of blood APOE4 neutrophils in cognitively impaired female *APOE4* carriers, which display increased IL-17 expression. This subset suppresses the induction of protective DAM through IL-17F interacting with microglial IL-17RA [289]. In contrast, the role of neutrophils in PD has not been directly investigated. The well-established link between peripheral inflammation and α-synuclein pathology suggests that the neutrophil-mediated neuroinflammation may also warrant exploration in this context [290].

Collectively, peripheral immune cells, including T cells, monocytes, NK cells, and neutrophils, actively infiltrate the brain parenchyma to modulate neuroinflammation in AD and PD through subset-specific and often disease-dependent mechanisms. T cells exert dual pathogenic and protective functions, whereas monocytes display distinct roles depending on the underlying proteinopathy. NK cells and neutrophils may contribute through mechanisms involving phagocytosis and inflammatory signaling, though their roles remain less comprehensively characterized in certain disease contexts. Together, these findings establish peripheral immune cells as integral regulators of neuroinflammation.

## Immune dysregulation and failure of resolution

Beyond excessive immune activation, immune dysregulation and failure of inflammatory resolution are closely associated with the persistence of chronic neuroinflammation in AD and PD. Under physiological conditions, an inflammatory response should transition into an active resolution phase that restrains further amplification, promotes the clearance of toxic materials and dead cells, and restores tissue homeostasis [291]. In chronic neurodegenerative diseases, however, inflammatory signaling persists, whereas the mechanisms required to terminate inflammation, clear pathological substrates, and support tissue repair gradually lose efficiency. This suggests that chronic neuroinflammation reflects not only excessive activation, but also impaired immune restraint, defective glial clearance, insufficient pro-resolving signaling, and loss of adaptive immune control.

A major mechanism contributing to the failure of resolution is the dysregulated immune checkpoint signaling. The PD-1/PD-L1 axis is a canonical inhibitory immune checkpoint pathway [292, 293]. Engagement of PD-1 by PD-L1 suppresses T cell receptor-driven proliferation and cytokine production, thereby maintaining peripheral tolerance and limiting immunopathology [292]. In AD, systemic PD-1 or PD-L1 blockade has been reported to mobilize peripheral immune responses, recruit monocyte-derived macrophages into the brain, and improve cognitive function [294, 295]. However, these findings have not been consistently reproduced across different AD mouse models [296]. By contrast, astrocytic PD-L1 can suppress neuroinflammation and preserve Aβ uptake by engaging microglial PD-1 [297], indicating that checkpoint signaling within the CNS may also be protective. In PD, peripheral T cell abnormalities are well documented, but early-to-mid-stage idiopathic PD does not uniformly display a classical CD8^+^ T cell exhaustion phenotype defined by PD-1, CTLA4, or LAG3 [298].

In addition to PD-1/PD-L1, the TGF-β pathway functions as a checkpoint-like regulatory axis. Under physiological conditions, TGF-β signaling sustains microglial homeostasis [299]. In AD, APOE4 impairs microglial response partly by promoting ITGB8–TGFβ signaling, which induces TGFβ-mediated homeostatic checkpoints and constrains the transition of microglia toward highly effective disease-responsive and phagocytic states [300]. In PD, TGFβ1 is induced in activated microglia and neurons in the 6-OHDA model, accompanied by downregulation of pro-inflammatory markers and induction of anti-inflammatory markers, supporting a restraining role for TGF-β signaling in microglial inflammatory activation [301]. Taken together, these findings suggest that dysregulated checkpoint signaling helps determine whether neuroinflammation in AD and PD can progress toward effective resolution or instead remains persistent and clearance defective.

Another major contributor to the failure of resolution is the defective glial clearance of pathological substrates and cellular debris. Under normal conditions, microglia and astrocytes engulf, degrade, and sequester abnormal proteins and damaged cellular material to maintain brain homeostasis [302, 303]. In chronic neurodegenerative diseases, however, this function gradually becomes inefficient. In the aged brain, neuron-derived proteins continuously transfer to and accumulate in microglia, with delayed degradation or aggregation, resulting in proteostatic imbalance, synaptic injury, and cognitive decline [304]. A terminally inflammatory microglial population has been detected accumulating in AD brains, which retains inflammatory features but has lost the capacity to efficiently handle Aβ [305, 306]. Impaired astrocytic clearance can also drive disease progression. Astrocytic αB-crystallin, which is highly expressed in PD, suppresses autophagy and impairs the clearance of α-synuclein aggregates, thereby worsening neuropathology in PD models [307]. Inflammatory stimulation suppresses uptake of α-synuclein fibrils by human microglia and disrupts phagosome maturation [308], while microglial α-synuclein accumulation promotes phagocytic exhaustion [309]. Therefore, chronic neuroinflammation in AD and PD reflects not only sustained inflammatory signaling, but also progressive collapse of glial substrate clearance.

Besides checkpoint dysfunction and impaired clearance, chronic neuroinflammation is further sustained by deficiency, mistiming, and dysfunction of specialized pro-resolving mediators. CSF from patients with mild cognitive impairment (MCI) and AD contains lower levels of pro-resolving mediators, including resolvin (Rv) D1, RvD4, NPD1, maresin 1, and RvE4, and increased levels of pro-inflammatory lipid mediators such as LTB_4_ and 15-HETE [310]. Functional studies further support the causal role of these lipid mediators. In AD models, combined RvE1 and lipoxin A4 treatment reduces neuroinflammation and Aβ pathology [311]. Intranasal delivery of a pro-resolving lipid mixture containing RvE1, RvD1, RvD2, maresin 1, and NPD1 improves memory performance, partly restores gamma oscillations, and suppresses microglial activation [312]. Maresin 1 also enhances Aβ42 uptake, and suppresses NF-κB activation and inflammatory mediator release [313].

Similar observations have been made in PD-related models. RvD1 reduces central and peripheral inflammation, preserves dopaminergic function, and delays motor impairment in experimental PD. RvD1 reduction in the CSF and plasma of early PD patients supports the translational relevance [314]. In vitro, both RvD1 and maresin 1 demonstrate protective effects in MPP^+^- and rotenone-toxicity models [315, 316]. These findings position defective specialized pro-resolving mediator signaling as a central mechanism underlying the failure of immune resolution in both AD and PD.

Failure of resolution is not solely confined to innate immunity. Impaired adaptive immune restraint, particularly defective Treg cell function, also contributes to chronic neuroinflammation. Adoptive transfer of Tregs into 3 × Tg-AD mice improves cognition and reduces Aβ deposition, whereas Treg depletion accelerates cognitive decline and alters microglial responses to amyloid deposits [317, 318]. In PD, CD28 agonist-induced Treg expansion suppresses early inflammatory responses, reduces activated T cells and CD11b^+^ microglia in the brain, and attenuates later dopaminergic neurodegeneration in A53T-α-synuclein models [319]. Likewise, adoptive transfer of α-synuclein-specific Tregs, which are regulatory T cells expanded in response to α-synuclein antigen presentation, more effectively suppresses pro-inflammatory microglia, reduces α-synuclein accumulation, and limits neuronal loss in MPTP-induced PD models [320]. These studies indicate that inadequate peripheral immune restraint is also involved in the failure of neuroinflammation resolution in AD and PD.

The progressive failure of inflammatory resolution in AD and PD is unlikely a result from a single process, but from the gradual loss of molecular, cellular, and anatomical conditions required to terminate inflammation and restore tissue homeostasis.

First, aging progressively impairs immune suppression and resolution within the CNS. Aged microglia show reduced phagocytic competence, downregulation of homeostatic genes, and decreased expression of receptors, such as TREM2, together with increased inflammatory programs [321]. Simultaneously, inhibitory regulatory pathways are altered with age. CD22 is upregulated in aged microglia, and its blockade restores a more homeostatic transcriptional state and improves cognition [322]. In AD-prone settings, APOE4 interacts with aging to blunt microglial surveillance, reduce P2RY12-dependent process motility, and promote the ITGB8-TGFβ-dependent checkpoint activity [300, 323].

Second, pathological protein aggregates can directly impair phagocytic degradative machinery. Aβ impairs nuclear translocation of TFEB (transcription factor EB), reduces expression of osteoclastogenesis-associated transmembrane protein 1, and weakens lysosomal acidification in microglia, thereby compromising Aβ degradation [324]. In PD, α-synuclein suppresses microglial autophagy through TLR4 and downstream p38/Akt-mTOR signaling, thereby amplifying neuroinflammation and neuronal injury, and this defect is further aggravated in the aged brain [325, 326].

Third, BBB disruption removes the anatomical containment required for local resolution of inflammation. An intact barrier helps preserve CNS immune compartmentalization, whereas its breakdown allows sustained entry of blood-derived proteins and peripheral immune signals that prolong inflammatory activity and prevent the restoration of immune quiescence. In AD, *APOE4* carriers show hippocampal and medial temporal barrier breakdown even before overt cognitive impairment [327]. In PD, human α-synuclein overexpression in transgenic mice induces vascular pathology, barrier leakage, and pericyte activation [328]. Once the barrier is compromised, the brain parenchyma becomes exposed to blood-derived proteins such as fibrinogen [329] and to inflammatory peripheral immune cells [330], both of which can drive neurotoxic microglial programs and further amplify neuroinflammation [331]. These processes reinforce one another, creating a self-perpetuating vicious cycle in which inflammation persists while clearance and repair progressively fail.

Overall, chronic neuroinflammation in AD and PD is more accurately conceptualized as a result of failed immune resolution rather than simply excessive immune activation. Dysregulated checkpoint signaling, defective glial clearance, impaired pro-resolving mediators, and loss of adaptive immune restraint, collectively contribute to a pathological state in which inflammation persists while clearance and repair mechanisms collapse. Future therapeutic strategies should therefore shift from nonspecific anti-inflammatory suppression toward restoration of endogenous pro-resolving pathways, enabling glial and immune cells to clear pathological proteins and cellular debris while limiting the chronic inflammatory responses that contribute to neurodegenerative damage.

## Similarities and differences in neuroinflammation between AD and PD

### Similarities

**Fig. 3:**
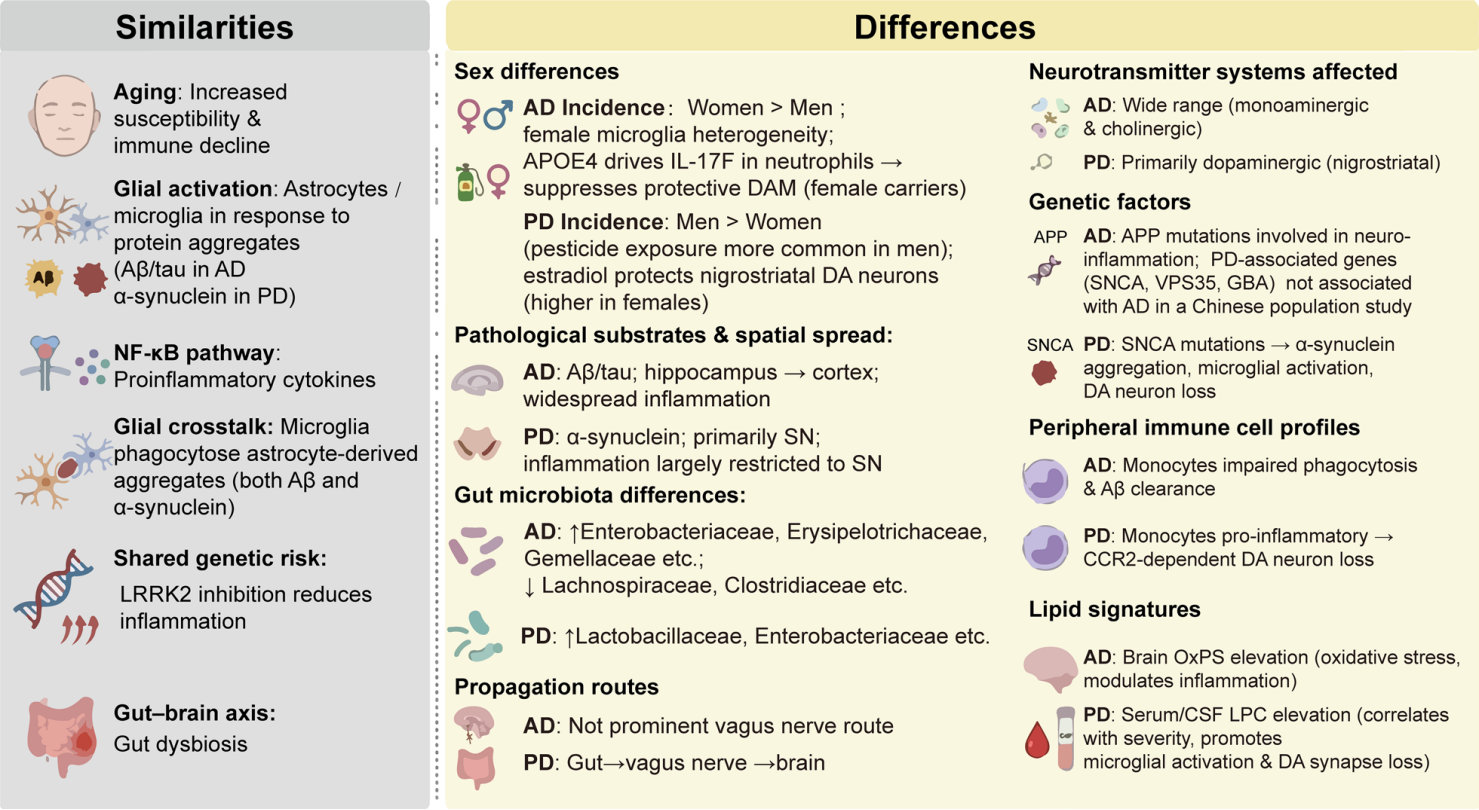
Similarities and differences in neuroinflammation-associated factors between AD and PD. AD and PD exhibit both commonalities and distinct features in the context of neuroinflammation. Similarities: Aging correlates with neuroinflammation development in both AD and PD. Pathological protein aggregates, including Aβ and tau in AD and α-synuclein in PD, accompany the activation of astrocytes and microglia. Long-term glial activation coincides with elevated inflammatory states, alongside continuous neuroinflammation progression. Shared genetic factors and the gut-brain axis present pathological relevance in both conditions. Differences: The two diseases display obvious distinctions in multiple dimensions potentially related to neuroinflammation: including (1) sex differences; (2) pathological substrates and spatial spread; (3) gut microbiota differences; (4) propagation routes; (5) neurotransmitter systems affected; (6) genetic factors; (7) peripheral immune cell profiles; (8) lipid signatures

As age-related neurodegenerative disorders, AD and PD exhibit numerous parallels in their neuroinflammatory signatures, reflecting the involvement of conserved pathophysiological pathways. A fundamental shared risk factor is aging, which significantly increases susceptibility to both diseases  [332, 333]. Aging is accompanied by progressive immune decline, in which dysregulated activation of innate immune pathways serves as a principal regulator of age-associated inflammation  [334]. This positions aging as a critical modulator of inflammatory responses in AD and PD (Fig. 3).

A core pathogenic mechanism common to both disorders is the activation of glial cells in response to pathological protein aggregates. Astrocytes and microglia recognize misfolded protein species and induce signaling cascades that launch and amplify neuroinflammation  [173, 335] (Fig. 3). Central to this process is the NF-κB pathway, whose activation represents a conserved hallmark in both disorders. NF-κB-mediated transcription drives the production of pro-inflammatory cytokines  [250, 336, 337], contributing to chronic pro-inflammatory responses in these two diseases.

Notably, emerging evidence reveals cooperative interactions between astrocytes and microglia in modulating aggregate clearance. In co-culture models, microglia actively recruit and phagocytose astrocyte-derived α-synuclein and Aβ aggregates, indicating that glial crosstalk not only propagates inflammation, but also critically regulates the removal of pathological protein deposits  [338] (Fig. 3). This dual role positions neuroinflammation as a dynamic process with both deleterious and adaptive functions in AD and PD pathogenesis. Moreover, neuroinflammation has also been shown to influence the clearance of disease-associated protein aggregates  [339]. Accordingly, identifying common inflammatory signaling pathways regulated by microglia and astrocytes in response to these pathological proteins may yield effective therapeutic strategies that target neuroinflammation across both disorders.

Beyond glial-mediated mechanisms, there are shared genetic determinants for neuroinflammation in AD and PD. Leucine-rich repeat kinase 2 (*LRRK2*) is the most prevalent genetic cause of familial PD  [340]. *LRRK2* mutations amplify astrocytic inflammatory responses to α-synuclein  [341]. Genetic variants in *LRRK2* have also been associated with increased AD risk  [342]. In addition, tau pathology, a cardinal feature of AD, has been observed in PD cases harboring *LRRK2* mutations  [340]. Notably, pharmacological inhibition of LRRK2 markedly reduces gliosis and neuroinflammation in both AD and PD mouse models  [343] (Fig. 3), highlighting its potential as a convergent therapeutic target. Together, these findings demonstrate that genetic regulators of inflammation are not confined to a single disease, but instead modulate common inflammatory cascades across neurodegenerative disorders.

In addition, dysregulation of the neuroimmune–peripheral immune crosstalk constitutes a unifying pathogenic theme in AD and PD  [259]. Both disorders are associated with gut microbiota dysbiosis  [344, 345] (Fig. 3), and FMT in preclinical models can lower the levels of pro-inflammatory proteins [346, 347]. Systemic infections, including infection by viral pathogens, can also fuel neuroinflammation relevant to AD or PD  [348]. Furthermore, chronic inflammatory gastrointestinal conditions, such as IBD, are potentially linked to increased risk of both AD and PD, although the causal relationship has not been fully clarified  [349]. These findings underscore the critical role of the gut–brain axis in transmitting peripheral inflammation to the CNS, establishing a bidirectional loop that amplifies neurodegeneration in AD and PD.

### Differences

Although AD and PD are both age-related neurodegenerative disorders, they exhibit different sex disparities in epidemiology and underlying mechanisms. Importantly, it is suggested that males and females exhibit distinct neuroinflammatory profiles with aging [350, 351], underscoring the important role of neuroinflammation in shaping the sex-specific disease trajectories. In AD, women account for two-thirds of all cases and have a higher lifetime risk of developing the disease (one in five) compared to men (one in ten) (Fig. 3). While debate persists as to whether this discrepancy can be attributed solely to the longer lifespan of women, evidence indicates that sex independently modulates risk factors and disease-driving mechanisms. Morphological analyses have revealed marked sex differences in microglial phenotypes: male brains display a uniformly ramified morphology, whereas female brains exhibit pronounced microglial heterogeneity (Fig. 3). Moreover, microglial density is higher in men than in women [350]. Furthermore, APOE4 drives IL-17F expression in neutrophils in a sex-dependent manner. The expressed IL-17F interacts with IL-17RA to suppress the induction of protective DAM, a process that occurs predominantly in female *APOE4* carriers [289] (Fig. 3). These results indicate that microglia- and neutrophil-mediated neuroinflammation may contribute to the sex differences in AD (Fig. 3). In PD, a distinct epidemiological pattern is observed, with men exhibiting consistently higher incidence and prevalence than women. This male predominance may be partly attributed to occupational exposures such as pesticides that are more common in men [352] (Fig. 3). Additionally, estradiol has been indicated to promote the survival of nigrostriatal DA neurons, implicating estrogen in the lower prevalence of PD in females [353] (Fig. 3). Both pesticides and estrogen are well-recognized regulators of neuroinflammation [354, 355], suggesting that neuroinflammation associated with these factors may mediate the sex differences observed in PD. Notably, intrinsic sex differences in baseline estrogen levels do not manifest as concordant sex-biased prevalence patterns across AD and PD. This discrepancy suggests that estrogen-mediated effects are context-dependent, varying according to the pathological substrates, vulnerable brain regions, and cellular mechanisms that define each disease.

Neurodegenerative processes in PD and AD follow distinct spatial trajectories. In PD, neuronal loss is predominantly localized to the SN, while AD pathology typically originates in the hippocampus and progressively extends to cortical regions, which become the principal sites of degeneration  [3, 356] (Fig. 3). The protein aggregates underpinning these disorders (α-synuclein in PD and Aβ/tau in AD) differ substantially in their synthesis, structural features, and functional consequences [357] (Fig. 3).

Current evidence indicates that the gut microbiota dysbiosis patterns associated with AD and PD are not fully consistent. In AD patients, gut microbial dysbiosis is characterized by elevated levels of the families *Enterobacteriaceae*, *Erysipelotrichaceae*, and *Gemellaceae*, as well as the genera *Akkermansia*, *Anaerotruncus*, *Collinsella*, *Bilophila*, *Escherichia-Shigella*, *Flavobacterium*, *Gemella*, *Lactobacillus*, and *Solobacterium* [358, 359]. In contrast, the families *Lachnospiraceae* and *Clostridiaceae*, together with the genus *Roseburia*, are decreased [358, 359] (Fig. 3). For PD patients, the abundance of *Lachnospiraceae* is significantly reduced relative to healthy controls, accompanied by markedly increased levels of *Lactobacillaceae*, *Enterobacteriaceae*, and *Enterococcaceae* [360] (Fig. 3). Given that gut microbiota plays important roles in neuroinflammation, it is hypothesized that the distinct alterations in gut microbiota are associated with neuroinflammation linked to AD and PD. However, the causal relationships in humans have not yet been established.

Both AD and PD are associated with olfactory dysfunction, suggesting that the olfactory bulb may be as a common and critical gateway for both conditions [361]. However, α-synuclein in PD can propagate from the gut to the brain via the vagus nerve (Fig. 3), whereas this pathway is not prominent in AD. The differences in pathogenic proteins and transmission routes may affect distinct cell types along the propagation pathways, thereby leading to differences in the molecular and cellular characteristics of neuroinflammation [362]. In addition to propagation pathways, the neurotransmitter systems targeted by pathological proteins in AD and PD also differ to some extent. In AD, Aβ and tau deposits disrupt a wide range of neurotransmitter systems, affecting both monoaminergic and cholinergic pathways  [363] (Fig. 3). Conversely, aggregated α-synuclein in PD principally impacts the DA system, reflecting the selective vulnerability of nigrostriatal neurons  [364] (Fig. 3). These divergent pathological substrates give rise to distinct neuroinflammatory signatures. In AD, inflammatory activity evolves dynamically across disease stages: it emerges in the neocortex during MCI and subsequently spreads throughout the brain, with the temporal cortex assuming a central inflammatory role in late-stage disease  [21] (Fig. 3). This widespread pattern is in sharp contrast with the SN-restricted pattern of neuroinflammation in PD  [21, 365] (Fig. 3). Such regional and temporal specificity highlights the potential for anti-inflammatory strategies tailored to the unique anatomical and chronological dynamics of each disorder, offering a promising avenue for targeted therapeutic intervention.

Beyond protein aggregates, genetic factors further differentiate the inflammatory landscapes of AD and PD (Fig. 3). Each disorder is linked to distinct susceptibility genes that shape the inflammatory processes in a disease-specific manner, although certain genes, such as *SNCA*, encoding α-synuclein, modulate neuroinflammation in both conditions. α-Synuclein is widely expressed throughout the brain [366]. Notably, in some AD cases, α-synuclein exhibits a broad topographic distribution, with the amygdala harboring the highest levels of α-synuclein inclusions  [367]. Moreover, the structural features of α-synuclein-positive aggregates vary across brain regions and frequently diverge from classical Lewy bodies, particularly in the hippocampus, highlighting context-dependent differences in aggregation patterns  [367]. Functional studies indicate that *SNCA* mutations promote α-synuclein aggregation, trigger microglial activation, and amplify inflammatory responses, ultimately contributing to DA neuron loss in the SN of PD  [365]. However, a case–control study in a Chinese population found that common and rare variants of PD-associated genes, including *SNCA*, *VPS35*, and *GBA*, which have been implicated in inflammation  [368–370], were not associated with AD  [371] (Fig. 3). In contrast, mutations in the *APP* gene, which are closely linked to AD, are involved in neuroinflammation [372, 373]. These findings suggest that while certain genetic elements engage shared neuroinflammatory pathways, others exert disease-selective regulatory roles in AD versus PD (Fig. 3).

In parallel, peripheral immune cells exhibit disease-specific functional profiles. Monocytes in AD display impaired phagocytic activity and reduced Aβ clearance capacity [273, 374] (Fig. 3). PD monocytes adopt a pro-inflammatory phenotype, contributing to DA neuron loss in a CCR2-dependent manner [260, 276]. CD8^+^ T cells in AD comprise functionally distinct subsets, including a protective CXCR6^+^ population restricting amyloid pathology [264] and a neurotoxic granzyme K-producing subset that promotes tau pathology and neuronal dysfunction [265]. In contrast, CD8^+^ T cells in PD appear to mediate direct neuronal cytotoxicity through MHC-I-dependent mechanisms [267], whereas the presence of functionally protective subsets has yet to be clearly validated.

Lipidomic analyses have revealed distinct lipid signatures intimately linked to neuroinflammation in the brain and blood that differentiate AD from PD. In the AD brain, levels of sphingomyelins and oxidized phosphatidylserine (OxPS) are significantly elevated (Fig. 3), whereas LPCs, such as LPC 18:2 and LPC 17:2, are decreased [375]. OxPS elevation is associated with lipid peroxidation under oxidative stress and plays a role in modulating inflammation [376, 377] (Fig. 3). At the blood level, higher concentrations of ganglioside GM3 and LPC 18:1 predict faster progression from MCI to AD, highlighting their potential as candidate biomarkers [378]. In PD, elevated LPC levels have also been detected in the serum and CSF of PD patients, correlating with disease severity [379], suggesting that LPC may also serve as a promising diagnostic or prognostic biomarker. Mechanistically, LPC promotes microglial activation and drives dopaminergic synapse loss, linking peripheral lipid alterations to PD-relevant neuroinflammation [379] (Fig. 3). However, longitudinal lipid signatures with PD progression remain less established, and PD-specific lipidomic alterations in peripheral biofluids are still poorly characterized [375]. Collectively, these findings indicate distinct lipid profiles in AD and PD: elevated OxPS in the AD brain and increased LPC in the periphery, both associated with neuroinflammation and disease progression.

## New research methods on neuroinflammation

### Glial cell imaging techniques

In both AD and PD, microglial and astrocytic activation are dynamic processes that vary across disease stages and brain regions. However, it remains unclear whether these glial responses are primarily neuroprotective or detrimental at a given time point. PET imaging of glial activation offers a unique opportunity to address this question in vivo. A pivotal molecular target is the 18 kDa translocator protein (TSPO), a mitochondrial membrane protein potentially upregulated in activated microglia and astrocytes [380, 381].

TSPO tracers, such as [^11^C]PK11195 and [^18^F]GE-180, have been used to investigate neuroinflammation in AD and PD. In AD, post-mortem correlative studies have shown that [^11^C]PK11195 uptake correlates with Aβ aggregates [382, 383], directly linking neuroinflammation to Aβ pathology. Enhanced [^18^F]GE-180 uptake has been observed in 4-repeat tauopathies and was markedly reduced following microglial depletion in a human tau P301S transgenic mouse model  [380, 384], validating its utility as a marker of microglia-specific neuroinflammation in 4-repeat tauopathies. To improve cell-type specificity, microglia-selective molecular targets that directly report their activation states have been developed. TREM2, which is predominantly expressed on microglia and essential for CNS homeostasis, represents a prime candidate. Human autoradiography studies have demonstrated elevated binding of a copper-64-labeled TREM2-targeting PET radiotracer in the cortex of AD patients  [385]. For astrocyte-specific imaging, monoamine oxidase B, which is highly expressed in reactive astrocytes—has emerged as a promising biomarker  [386]. Oxidase B–targeted PET imaging has detected increased astrocytosis in AD patients  [386, 387], highlighting its potential as a marker of astrocyte-mediated neuroinflammation. In chronic PD animal models, [^11^C]PK11195 PET studies showed only a modest increase in tracer uptake, despite robust microglial IBA1 (ionized calcium-binding adaptor molecule 1) staining [388]. This discrepancy likely reflects the tracer’s low signal-to-noise ratio and limited sensitivity to region-specific inflammation in the SN. One contributing factor is the broad cellular expression of TSPO, which is present not only in microglia but also in astrocytes and endothelial cells, some of which may become activated during pathology and thus confound PET signal interpretation.

In summary, glial cell PET imaging has advanced our understanding of neuroinflammation in AD and PD by enabling in vivo assessment of glial activation. Nevertheless, its practical utility is limited by suboptimal signal quality, limited target specificity and occasional inconsistency of findings. Beyond microglia and astrocytes, OLG lineage cells also contribute to neuroinflammation in these diseases. Therefore, there is a pressing need to develop specific tracers that can detect OLG lineage cell-mediated neuroinflammation. Such tools would complement existing radiotracers targeting multiple cell types—including microglia, astrocytes, and neurons—and provide a more comprehensive view of neuroinflammatory dynamics throughout the course of AD and PD.

### Transcriptomics analysis

Transcriptomic technologies, from bulk RNA sequencing (bulk RNA‑seq) to single‑cell and single‑nucleus RNA sequencing (scRNA‑seq/snRNA‑seq), enable unbiased, genome-wide gene expression profiling in brain tissues from AD and PD  [389–391], yielding novel insights into the molecular underpinnings of neuroinflammatory responses and establishing a foundation for the identification of potential therapeutic targets.

Bulk RNA-seq has been instrumental in delineating the molecular signatures of neuroinflammation in AD and PD. In AD, comparative transcriptomic analyses of cortical tissue revealed a pronounced upregulation of pro-inflammatory cytokines in individuals at the earliest disease stage relative to middle-aged controls  [348], suggesting that neuroinflammation precedes clinical symptoms and may define a potential “window of opportunity” for early intervention. Post-mortem transcriptomic studies further identified marked alterations in DAM gene expression in AD  [392]. In PD, transcriptome analysis of the nigrostriatal region uncovered more than 300 differentially expressed genes, with the most significantly enriched pathways centered on “protein folding” and “neurotransmitter transport”  [393]. These findings underscore the utility of bulk RNA-seq for pinpointing dysregulated genes and signaling cascades within affected brain regions. However, bulk RNA‑seq cannot resolve cell‑type‑specific transcriptional differences, which is a major limitation given the cell‑specific nature of neuroinflammatory responses.

To address this limitation, scRNA-seq/snRNA-seq provides gene expression profiles at the single-cell level, enabling the detection of rare, inflammation-associated cell subpopulations and cross-disease phenotypic parallels between AD and PD  [202, 210]. For instance, snRNA-seq analysis of astrocytes from post-mortem brains of vascular dementia patients identified a distinct subpopulation marked by high expression of interferon- and neuroinflammation-related transcripts  [210]. Remarkably, this subset exhibited transcriptional overlap with neuroinflammatory astrocytes previously characterized in AD mouse models  [210, 394], suggesting that astrocyte-mediated inflammatory pathways are conserved across neurodegenerative diseases. In PD, snRNA-seq of post-mortem midbrain tissue from idiopathic PD patients revealed a subgroup of astrocytes with high *CD44* expression, accompanied by microglia displaying increased pro-inflammatory cytokines  [202]. This finding supports a role of astrocyte–microglia crosstalk in shaping neuroinflammation in PD, particularly within disease-relevant brain regions.

Despite these advances, single-cell studies have also revealed discordant findings regarding the role of microglia in PD. A snRNA-seq study of the SN and cortex reported that microglial gene expression signatures are strongly linked to genetic risks for AD, but not for PD  [221]. This observation challenges the conventional view that microglia-mediated neuroinflammation is a primary contributor in both disorders, raising the possibility that neuroinflammation in PD may be a secondary event rather than an initial pathogenic trigger. These discrepancies likely arise from sample and methodological differences, highlighting the need for standardized protocols and cross-cohort validation in AD/PD transcriptomics. To resolve inconsistencies and clarify disease-specific mechanisms, a multidisciplinary and integrative approach including artificial intelligence-assisted analysis is essential. In particular, transcriptomic data should be combined with functional assays to validate the biological relevance of identified genes and pathways for AD and PD.

### iPSCs and organoids

A key question in AD and PD is whether human glial cells exhibit disease‑specific activation states that cannot be recapitulated in animal models. iPSC technology, first described by Takahashi and Yamanaka  [395], allows the generation of specialized neural cell types from somatic cells. These cells can be exposed to pathological stimuli such as Aβ, α-synuclein, or LPS, and be used in neuron–glial co-culture systems  [396]. In AD-relevant contexts, LPS or Aβ induces increased levels of pro-inflammatory factors including IL-1β, IL-6 and TNF-α [397, 398]. In PD‑related conditions, human iPSC‑derived microglia from idiopathic PD patients, cultured in 2D and compared with age‑matched healthy controls, exhibit significantly elevated secretion of IL-1β and IL-10 upon LPS stimulation [398, 399]. These models circumvent cross-species differences and allow targeted analysis of inflammatory responses in specific glial subtypes. However, standard iPSC cultures lack three‑dimensional tissue architecture and vascularization, limiting their ability to recapitulate in vivo cellular interactions.

To address these shortcomings, human brain organoids (HBOs) have been developed as three-dimensional models that more closely approximate brain tissue structure  [396, 398, 400]. A notable technical advance is the generation of immunocompetent HBOs via xenotransplantation into immunodeficient mice, enabling the investigation of human microglia within a vascularized in vivo environment [401]. Human microglia residing in xenografted immunocompetent HBOs retain human-specific transcriptomic signatures and exhibit dynamic, in vivo-like behaviors [401], demonstrating the utility of HBOs for investigating neuroinflammation. In the specific context of AD, APOE4 aggravates synapse loss and neurodegeneration in cerebral organoids derived from patient iPSCs [402]. Moreover, a recent study established a human pluripotent stem cell-derived vascularized neuroimmune organoid model that recapitulates multiple sporadic AD pathologies including Aβ/tau aggregates and neuroinflammation upon exposure to patient brain extract and enables evaluation of efficacy and safety of AD drugs, such as lecanemab [403]. For PD, human midbrain organoids with *GBA1* defects and *SNCA* perturbations recapitulate key α-synuclein pathologies and Lewy body–like inclusions [404]. Midbrain organoids derived from human iPSCs can integrate into striatal circuits and rescue motor function in a PD mouse model [405]. Studies have also developed human striatal‑midbrain assembloids that recapitulate PD‑associated nigrostriatal circuits, reproduce α‑synuclein propagation and dopaminergic impairment, and faithfully mirror patient‑derived transcriptional signatures to support PD mechanistic research and drug screening [406].

Recent progress in long-term live light-sheet microscopy enables continuous tracking of HBOs development—capturing tissue morphology, subcellular features, and cellular behaviors over weeks  [407]. This imaging strategy can be employed to track neuroinflammatory dynamics in AD and PD models: in vitro by monitoring glial activation following exposure to Aβ or α-synuclein; and in vivo by visualizing glial responses within xenografted organoids residing in a living brain. However, current organoid systems lack long-range functional neural circuits [408], a circuit gap for modeling chronic neurodegenerative disorders such as AD and PD. Optimized systems should enable long-term, dynamic monitoring of inflammatory processes across defined brain regions, specific neural cell subsets, and peripheral interactions.

## Advances in therapeutics targeting neuroinflammation

With chronic neuroinflammation now recognized as a key contributor of pathology in AD and PD, therapeutic strategies are shifting from broad immunosuppression toward precision immunomodulation. This section reviews the leading approaches targeting neuroinflammation, including cytokine-directed biologics, microglial phenotype modulation, and nutritional interventions and current clinical trials. We also examine the scientific rationale, translational challenges, and potential for disease modification offered by each strategy in these neurodegenerative disorders.

### Anti-inflammatory cytokine therapy

#### TNF-α inhibitors

TNF-α is a well-established and extensively studied neuroinflammatory target in AD and PD. Aberrant TNF-α elevation is indicated to contribute to cognition dysfunction, increased tau phosphorylation, and neurodegeneration, highlighting its central role in disease progression [409, 410]. In the 5 × FAD mouse model of AD, peripheral administration of XPro1595, a selective soluble TNF inhibitor, reduces neuroinflammation, modulates brain immune cell profiles, lowers hippocampal Aβ plaque burden, and rescues impaired long-term potentiation. These results underscore the therapeutic promise of precise modulation of TNF signaling in mitigating AD pathological and synaptic impairments [411]. A recent phase II trial of XPro1595 reported reduced neuroinflammation and positive cognitive trends in early-stage AD patients exhibiting a high inflammatory burden without associated amyloid-related imaging abnormalities (ARIA) [412]. Nevertheless, verification of efficacy and safety in larger-scale, controlled clinical trials remains imperative.

#### IL-1 pathway inhibitors

The IL-1 cytokine family, notably IL-1β, serves as a principal downstream effector of NLRP3 inflammasome activation [413]. Elevated IL-1β levels have been implicated in the pathogenesis of both AD and PD [153], positioning IL-1 signaling as a compelling therapeutic target. Inhibition of IL-1 signaling improves cognitive performance and reduces tau pathology in a model of AD [414]. Inhibitors, such as Anakinra, an IL-1 receptor antagonist, attenuated neuroinflammation and neuronal injury, demonstrating efficacy in a mouse model of ischemic stroke [415]. A major advantage of targeting the IL-1 pathway is its upstream position within inflammatory cascades, which confers the potential for broad modulation of neuroimmune responses. Moreover, the existence of clinically approved agents, like Anakinra, facilitates drug repurposing opportunities for neurodegenerative diseases.

#### IL-6 and IL-6 receptor (IL-6R) inhibitors

IL-6 is a key pro-inflammatory cytokine with important roles in the neuroimmune system. It signals through soluble or membrane-bound IL-6R to activate the JAK-STAT pathway, thereby promoting chronic inflammation. Therapeutic agents, such as tocilizumab (an anti-IL6R monoclonal antibody) have been indicated to show improvements in clinical populations of encephalitis  [416]. It is speculated that these improvements are likely mediated by modulation of neuroimmune pathways, including BBB permeability, microglial activation, and hypothalamic–pituitary–adrenal axis regulation. Although the clinical application of these agents in AD and PD remains investigational, emerging evidence suggests their potential to alleviate neuroinflammation-linked depressive symptoms and cognitive impairment in these disorders [417].

#### IL-17 and IL-23 axis inhibitors

The IL-23/IL-17 axis is a central pathway in pathogenic inflammation and is gaining recognition for its emerging role in neurodegenerative processes. IL-23, produced by innate immune cells such as dendritic cells, is essential for the expansion and stabilization of Th17 cells, which are the principal source of IL-17  [418, 419]. Although this axis is well established in autoimmune disorders, such as multiple sclerosis, accumulating evidence supports its involvement in the pathogenesis of AD and PD  [419, 420]. Therapeutic agents targeting specific components of this pathway, such as IL-23p19 subunit blockers (e.g., guselkumab), have demonstrated clinical efficacy in ulcerative colitis [421], indicating the potential of IL17/IL23 in inflammatory associated brain diseases such as AD and PD although direct clinical evidence in these two neurodegenerative conditions remains limited.

Although compelling biological rationales support targeting cytokines therapeutically in AD and PD, substantial barriers impede clinical translation. Poor BBB penetration can restrict CNS bioavailability, diminishing the effectiveness of anti-cytokine therapies. Consequently, specialized delivery strategies are needed to overcome this obstacle. One emerging approach involves nanobodies, camelid-derived single-domain antibodies (VHH) of approximately 15 kDa [422]. Their small size and absence of an Fc region confer advantages in BBB crossing. Another promising strategy is ICAM-directed pulmonary leukocyte nanocarrier system to deliver drugs to the injured brain [423]. Therefore, combining the aforementioned techniques to further optimize drug delivery systems is also an important aspect of therapy targeting inflammatory factors.

Based on the above, treatment strategies targeting inflammatory factors hold significant clinical value. Nonetheless, definitive confirmation of efficacy and safety in larger, controlled trials remains essential before such approaches can be widely adopted. Additionally, systemic administration of anti-cytokine antibodies raises concerns about compromised host defense and increased susceptibility to infections, especially in elderly patients. Furthermore, the chronic progression of AD and PD may necessitate long-term treatment, yet comprehensive data on the safety and tolerability of extended therapy remain scarce. In summary, although anti-cytokine therapy is mechanistically well justified, its successful clinical translation will likely depend on the development of optimized CNS delivery systems, refined patient stratification strategies, and carefully designed combination regimens.

### Potential therapeutics targeting microglial neuroinflammation

In contrast to broad-spectrum immunosuppression, which can impair the brain’s innate defense mechanisms, a more refined therapeutic strategy has attracted considerable interest. This approach seeks to modulate microglial phenotype and function, specifically by steering them from a chronic pro-inflammatory state toward a protective, homeostatic, or anti-inflammatory profile  [21, 424]. The goal of this strategy is not to abolish microglial activity altogether, but to reprogram these cells into a protective phenotype marked by enhanced phagocytic capacity, release of neurotrophic factors, and secretion of anti‑inflammatory cytokines that promote tissue repair and inflammation resolution [425]. This phenotypic reconfiguration presents a promising way to break the self‑reinforcing cycle of neuroinflammation, with potential to slow disease progression and even foster neural restoration in AD and PD  [297, 426]. Key targets for microglial reprogramming include the following.

#### TREM2 agonists

TR﻿EM2 is a microglial surface receptor critical for detecting lipid damage and regulating microglial metabolism, survival, and phagocytosis [427, 428]. Genetic evidence has firmly established that certain *TREM2* variants confer increased AD risk  [429], highlighting its important role in disease pathogenesis. In preclinical models, agonistic antibodies that activate TREM2 have demonstrated therapeutic efficacy: they drive microglial clustering around Aβ plaques, boost phagocytic clearance, attenuate neuroinflammation, and improve cognitive outcomes  [430–432]. Collectively, these findings position TREM2 as a leading therapeutic target for AD, with the potential to modulate microglial function to counteract neurodegeneration.

#### PPARγ agonists

Peroxisome proliferator-activated receptor-gamma (PPARγ) is a nuclear receptor implicated in regulating the expression of pro-inflammatory genes in glial cells  [433]. Pioglitazone, a PPARγ agonist, has demonstrated anti-inflammatory and neuroprotective effects in preclinical models  [434, 435]. However, the clinical trial outcomes of PPAR agonists in neurodegenerative diseases remain not fully inconsistent, largely because certain studies have reported very limited efficacy. More recent evidence points toward a potentially beneficial effect of PPAR activation [436]. This variability is likely due to several factors, including differences in specific agonist properties and patient populations.

#### CSF1R inhibitors

CSF1R is essential for microglial survival. Transient pharmacological inhibition of CSF1R with small molecules, such as PLX3397, leads to substantial depletion of resident microglia  [437]. Upon treatment cessation, the brain undergoes microglial repopulation by newly generated cells, effectively resetting the local immune milieu  [438]. This approach has produced striking benefits in neurodegenerative disease models. In AD, it promotes Aβ plaque clearance, reduces tau pathology, and improves cognitive performance [439–442]. Nevertheless, it exerts no impact on microglial response and DA neuron degeneration in a rat model of PD  [443]. These findings position microglial depletion followed by repopulation as a bold yet promising therapeutic strategy, albeit one likely confined to certain neurodegenerative conditions.

In alignment with these findings in animal studies, recent work from Peng’s group has demonstrated that CSF1R-mediated microglial replacement offers a novel and effective approach for CNS disorders. Notably, microglial replacement has shown therapeutic potential in patients of adult-onset leukoencephalopathy with axonal spheroids and pigmented glia [444, 445], highlighting its potential as a transformative modality for neurodegenerative diseases such as AD and PD.

#### ﻿Nicoti﻿namide adenine dinucleotide (NAD^*+*^) supplementation

NAD^+^ is a fundamental molecule regulating cellular metabolism, mitochondrial function and cell survival [446, 447]. Declining NAD^+^ levels have been observed in aging and neurodegeneration, which are potentially associated with mitochondrial dysfunction and pro-inflammatory microglial activation [447, 448]. Studies have revealed that NAD^+^ notably attenuates microglial activation and downregulates the expression of pro-inflammatory factors elicited by chronic cerebral hypoperfusion. In vitro assays further validated that NAD^+^ exerts protective effects on BV2 microglial cells against hypoxia-induced neuroinflammation, mitochondrial damage, and reactive oxygen species overproduction [449]. Nicotinamide mononucleotide, a direct precursor of NAD^+^, combined with the PARP1 inhibitor PJ-34, can alleviate LPS-mediated mitochondrial damage in microglial cells [450]. These findings indicate that NAD^+^ regulates microglia-mediated neuroinflammation.

Accumulating evidence has validated the involvement of NAD⁺ in modulating neuroinflammation in the context of AD and PD. Reduction of NAD^+^ levels accompanied by upregulation of inflammation markers is observed in AD mouse brain. Supplementation with the NAD^+^ precursor nicotinamide riboside effectively elevates NAD^+^ content, decreases pro-inflammatory cytokine expression and mitigates microglial activation in AD mice. In addition, cell experiments demonstrate that nicotinamide riboside ameliorates microglial neuroinflammation through the cGAS-STING pathway [451]. Similarly, the mean NAD⁺ levels are significantly lower in PD subjects compared with controls [452]. Nicotinamide, the amide form of niacin and an important precursor of NAD^+^, can reduce MPTP-induced inflammatory responses in the striatum and SN, contributing to the improvement of PD-associated behavior [453].

These preclinical findings have been extended clinically. An early pilot study investigated 10 mg daily oral stabilized NADH (an important NAD^+^ precursor) in 17 patients with AD-related dementia. After 6–12 weeks, the treatment was well-tolerated and associated with significant cognitive improvements [454]. A double-blind phase I clinical trial in PD patients found that oral nicotinamide riboside (1000 mg/day) is well-tolerated, increases cerebral NAD^+^, alters brain metabolism, and is associated with mild clinical improvement. Nicotinamide riboside enhances NAD^+^ metabolism, upregulates cellular function, and reduces inflammation, further confirming its promising neuroprotective value [455]. Nevertheless, several clinical trials have failed to yield ideal therapeutic outcomes, and some relevant clinical investigations are still in progress [447].

### Anti-inflammatory diet

Nutritional interventions offer an accessible, non-pharmacological strategy for modulating systemic inflammation. The gut–brain axis is increasingly recognized as a pivotal conduit in AD and PD, since dietary patterns can influence systemic inflammation, gut microbiota composition, and ultimately CNS homeostasis  [456, 457]. This connection between diet, the gut–brain axis, and neurodegeneration underscores how environmental factors, diet in particular, impact CNS health, and highlights the need to investigate specific dietary approaches for their potential to modulate neuroinflammation in these diseases.

Among nutritional strategies, the Mediterranean diet and ketogenic diet have been the most extensively studied  [458–460]. The Mediterranean diet is characterized by high intake of fruits, vegetables, whole grains, olive oil, and fish, and is abundant in polyphenols and omega-3 polyunsaturated fatty acids, compounds with well-established anti-inflammatory and antioxidant properties. Observational studies consistently link adherence to this dietary pattern with a reduced risk of AD and PD, as well as slower cognitive decline  [461, 462]. Proposed mechanisms include rebalancing pro- and anti-inflammatory responses and fostering beneficial gut bacteria that generate anti-inflammatory metabolites, such as short-chain fatty acids  [463]. However, it remains uncertain whether healthy dietary patterns derived from Western populations are directly applicable to non-Mediterranean groups, including the East Asian population, given substantial differences in dietary habits, genetics, and metabolic phenotypes.

Recently, the Jiangnan Diet has been proposed as a regional counterpart to the Mediterranean diet [464]. This dietary pattern, rooted in the traditional eating habits of the Yangtze River Delta region, is characterized by long-standing consumption among its native inhabitants. Epidemiological observations indicate that individuals in this region experience a lower incidence of MCI and enjoy greater average life expectancy compared with people in other areas  [465, 466]. The Jiangnan Diet shares several hallmarks with the Mediterranean diet, particularly a high intake of vegetables and moderate consumption of fruit. For a comprehensive discussion of its composition and health implications, readers are referred to the review by Wang et al*.*  [465]. In parallel, the ketogenic diet, defined by high fat and very low carbohydrate intake, shifts energy metabolism toward reliance on ketone bodies. Ketones not only serve as an efficient alternative fuel for neurons, but also exhibit anti-inflammatory properties [467–469], indicating therapeutic potential in these disorders targeting neuroinflammation.

Nutritional interventions appear to hold promise for disease prevention and may serve as valuable adjuncts to pharmacological therapies. However, there is currently no evidence that they can delay disease progression in AD or PD. This limitation likely stems from several factors. First, clinical diagnosis of AD/PD typically occurs after substantial pathological changes and neuronal loss have already taken place. Consequently, nutritional strategies cannot reverse established neuropathology, but mainly act to modulate pro-resolving neuroinflammation. Second, many bioactive nutritional components, such as polyphenols, face the challenge of poor BBB penetration, resulting in low bioavailability and diminished efficacy within the CNS.

Taken together, the wide range of therapeutic strategies reviewed here, from advanced biologic agents to nutritional interventions, constitutes a multifaceted armamentarium for combating neuroinflammation in neurodegenerative diseases. Each approach offers unique strengths but also entails distinct limitations. Biologic therapies afford high target specificity within well‑characterized pathogenic cascades, yet encounter challenges, such as high cost, risk of systemic immunosuppression, and limited BBB penetration. Microglial reprogramming strategies seek to reinstate homeostatic function rather than merely suppress activation, embodying a finely tuned therapeutic paradigm. However, achieving precise control of microglial activity in diseased brains in a spatial and temporal manner remains technically demanding. As an alternative route to restoring CNS homeostasis, microglial replacement has recently demonstrated efficacy in a clinical trial  [444], offering a promising avenue to direct microglial modulation.

The future advancement of neuroinflammatory therapeutics will hinge on progress in several critical domains. First, validated biomarkers, such as blood-based neuroinflammation-associated molecule signatures or PET ligands capable of imaging multiple glial cell populations, are essential for identifying individuals with active neuroinflammatory phenotypes who are most likely to benefit from targeted interventions. Second, therapeutic windows must shift earlier, toward the prodromal phase of disease, before irreversible neuronal loss occurs and neuroinflammatory cascades become self-sustaining. Third, pinpointing the origins of neuroinflammation and eliminating inflammation-propagating triggers may be critical for more effective control of CNS inflammation. Peripheral tissues and organs, particularly the gut, exert complex, multidirectional influences on CNS homeostasis. For example, the gut produces both pro- and anti-inflammatory mediators; gut-derived α-synuclein can fuel inflammatory responses during PD pathology [470], whereas microbial metabolites such as *N*-acetyl-L-lysine have been shown to exert anti-inflammatory effects in animal models of multiple sclerosis  [471]. Precision modulation of these peripheral systems could influence the trajectory of systemic and neuroinflammation. Finally, integrated therapeutic strategies that concurrently target multiple pathological axes (e.g., intestinal barrier dysfunction, neuroinflammation, BBB breakdown, and protein misfolding) may yield synergistic benefits. Although translational hurdles persist, particularly in drug delivery and the design of long-term clinical trials, the strategic modulation of neuroinflammation represents a compelling path toward disease-modifying treatments for AD and PD.

### Potential therapeutics targeting gut microbiota

Gut microbiota dysbiosis is increasingly recognized as a contributor to neuroinflammation and amyloid pathology in AD through the microbiota–gut–brain axis [472]. In AD, oral administration of *F. prausnitzii* strains isolated from healthy humans improved memory deficits in an Aβ-injected mouse model, with pasteurized bacteria showing efficacy in long-term memory tests [473]. Mechanistically, gut dysbiosis activates the C/EBPβ/AEP signaling pathway in the intestine and brain, promoting amyloidogenesis and neuroinflammation. Antibiotic treatment or prebiotic intervention suppressed this pathway, reduced Aβ pathology, and rescued cognitive function in 5 × FAD mice [474]. Dietary interventions such as probiotics (*Lactobacillus* and *Bifidobacterium*), prebiotics (galacto-oligosaccharides, fructo-oligosaccharides), and synbiotics, have been shown to modulate gut microbiota composition, enhance short-chain fatty acid production, and improve cognitive outcomes in preclinical models, though clinical evidence remains preliminary [475].

In PD, antibiotic exposure, particularly to broad-spectrum agents and antifungals, is associated with elevated PD risk, with peak susceptibility occurring 10–15 years prior to diagnosis [476]. Clinical trials of FMT demonstrate significant improvements in gastrointestinal symptoms and partial alleviation of motor dysfunction in PD patients compared to placebo, with no severe adverse effects reported [477]. The gut microbiome in PD is profoundly regulated by dietary patterns: higher Healthy Eating Index (HEI-2015) scores and fiber intake correlate with enriched anti-inflammatory butyrate producers, while elevated added sugar consumption associates with pro-inflammatory taxa [478]. Meta-analyses confirm these microbial signatures are reproducible across diverse populations and may serve as early biomarkers, as similar dysbiosis patterns are detected in prodromal stages and genetically at-risk individuals (e.g., *GBA1* variant carriers) [479, 480]. Mechanistically, the gut-brain axis contributes to PD pathology, with evidence suggesting pathological α-synuclein aggregation may originate in the enteric nervous system and propagate to the CNS via the vagus nerve [481, 482]. Collectively, these findings highlight microbiome-targeted strategies as promising therapeutic avenues for modifying PD progression.

### Current development of specific clinical trials for AD

The clinical success of Aβ monoclonal antibodies has validated the amyloid hypothesis and provided the first disease-modifying therapies for AD [483, 484]. However, these agents also revealed a critical interplay between amyloid clearance and neuroinflammation, most notably through ARIA—a dose-limiting adverse event characterized by vasogenic edema or hemosiderin deposition that occurs with all amyloid-removing antibodies [485, 486]. ARIA arises from antibody binding to vascular amyloid, activating perivascular macrophages and disrupting BBB integrity. Its incidence is influenced by antibody dose and *APOE* ε4 genotype, and has historically limited therapeutic exposure [487].

A transformative solution emerged from engineering the route of brain entry. Pizzo and colleagues developed an antibody transport vehicle (ATV) targeting the transferrin receptor with asymmetrical Fc mutations that preserve effector function while redirecting CNS delivery [488]. Unlike conventional anti-Aβ antibodies that enter via perivascular spaces, concentrating at cerebral amyloid angiopathy-laden arteries, ATV-mediated transport occurs through capillaries where transferrin receptor expression is highest, enabling broad parenchymal distribution while avoiding arterial accumulation. In 5 × FAD mice, ATVV^cisLALA^: Aβ nearly eliminated ARIA-like lesions despite achieving higher brain exposure, demonstrating that biodistribution engineering can dissociate efficacy from vascular side effects [488, 489].

Complementing delivery innovations, TREM2 agonists, which enhance microglial phagocytosis, attenuate neuroinflammation [60, 105, 427, 490]. In principle, it is suggested that combining TREM2 agonists with anti-Aβ therapy may augment amyloid removal while dampening perivascular inflammation, though clinical validation awaits. *APOE4* carriers face higher ARIA risk due to inherent BBB vulnerability, supporting genotype-guided dosing [327, 486]. Effective disease modification likely requires targeting multiple nodes: removing Aβ, modulating immune responses (TREM2, NLRP3), and intercepting tau pathology [491]. Thus, the evolution of AD-specific therapies is increasingly defined by their ability to precisely calibrate the neuroimmune response, transforming amyloid clearance into a finely tuned restorative process.

### Current development of specific clinical trials for PD

Immunotherapies targeting α-synuclein have emerged as leading disease-modifying strategies for PD, aiming to extinguish the driver of chronic neuroinflammation [364, 492]. Over the past five years, both passive and active immunization approaches have advanced through clinical testing.

#### Passive immunization

Prasinezumab, a monoclonal antibody targeting the C-terminus of aggregated α-synuclein, was evaluated in the phase II PASADENA study [493]. While the primary endpoint was not met, prespecified subgroup analyses revealed that prasinezumab significantly slowed motor progression, reducing MDS-UPDRS Part III decline by 40%–64%, in patients with rapidly progressing disease or those on stable oxidase B inhibitor treatment [494]. These findings highlight the importance of patient stratification by progression rate and have informed the ongoing phase IIb PADOVA trial (NCT04777331) [492]. By contrast, cinpanemab, an N-terminal-targeting antibody, failed to demonstrate efficacy in the phase II SPARK study despite robust target engagement, underscoring the critical role of epitope specificity [495, 496]. Lu AF82422, an antibody targeting α‑synuclein,showed signals of efficacy in less impaired patients, with reduced brain volume loss and decreased CSF neurofilament light chain levels, supporting further evaluation in phase III [492, 497].

#### Active immunization

PD01A, a peptide-based vaccine targeting the C-terminus of α-synuclein, demonstrated safety and sustained immunogenicity over 3.5 years of follow-up in a phase I study [498]. Antibodies induced by PD01A selectively recognized oligomeric α-synuclein, and post-hoc analysis revealed a 51% reduction in CSF oligomeric α-synuclein in high-dose recipients, the first evidence of target engagement for active immunotherapy in PD [498]. More recently, UB-312, a vaccine platform utilizing synthetic T-helper peptides, successfully overcame immune tolerance, generating anti-α-synuclein antibodies detectable in both serum and CSF [499, 500]. In a phase I study, 12/13 patients achieved seroconversion, and those with detectable CSF antibodies showed significant reductions in α-synuclein seeding activity (measured by α-synuclein seed amplification assay) and improvements in MDS-UPDRS Part II scores, directly linking antibody exposure to clinical benefit [500].

#### Targeting the GBA pathway

GBA1 mutations, the most common genetic risk factor for PD, cause glucocerebrosidase (GCase) deficiency, impaired lysosomal function, and secondary α-synuclein accumulation, establishing a bidirectional pathogenic loop that amplifies neuroinflammation [501, 502]. Ambroxol, a small molecule chaperone that enhanced GCase activity, increased GCase levels   and increased CSF α-synuclein in a phase II trial, though clinical efficacy requires confirmation [503]. Gene therapy approaches delivering functional *GBA1* via adeno-associated viral vectors have shown preclinical promise, restoring GCase activity and attenuating microglial activation in mouse models [504, 505].

In summary, PD-specific strategies target α-synuclein to remove the inflammatory trigger and restore GBA-mediated lysosomal function to correct cellular vulnerability, both aiming to extinguish the driver of neuroinflammation rather than suppress downstream consequences. Emerging biomarker-defined patient subgroups and target engagement data suggest that these upstream interventions translate into measurable biological effects. Future integration of α-synuclein clearance with direct modulation of microglial responsiveness may offer synergistic benefits, addressing both the inducer (α-synuclein) and the effector (glial cells) of neuroinflammatory pathology in PD.

### Challenges, failures, and unresolved questions in clinical translation

Despite robust preclinical rationale, nearly all large-scale trials of anti-inflammatory therapies for AD and PD have failed, including nonsteroidal anti-inflammatory drugs (ADAPT, ASPREE, rofecoxib) and minocycline [506–512]. These failures reveal significant limitations: delayed intervention (treatment after irreversible neuronal loss), poor BBB penetration, single-target inhibition in a redundant network, lack of biomarker guided patient stratification, dual roles of inflammation (broad suppression eliminates protective phagocytosis), and suboptimal dosing [101, 513–518].

These collective failures underscore the need for a fundamental paradigm shift from broad, non‑specific anti‑inflammatory approaches toward a precision neuroimmunology framework that embraces the complexity of CNS immune responses. This transition rests on several interrelated priorities. First, therapeutic intervention must be deployed earlier in the disease continuum, targeting biomarker‑defined prodromal populations before irreversible neuronal loss has occurred. Second, achieving this goal requires optimized CNS delivery—engineered biologics (e.g., transferrin receptor‑targeted transport vehicles) and brain‑penetrant small molecules that can achieve therapeutically relevant concentrations within the brain parenchyma. Third, given the redundant architecture of neuroinflammatory networks, effective modulation will likely demand multi‑targeted pathway inhibition, combining upstream regulators (NLRP3 inflammasome, TREM2) with downstream effector nodes. Fourth, such strategies can only succeed if coupled with biomarker‑guided patient stratification, using CSF markers (sTREM2, YKL‑40, GFAP) and next‑generation PET tracers capable of distinguishing discrete glial activation states to identify those individuals most likely to benefit.

## Conclusions

This review has systematically explored the contributory and amplifying roles of neuroinflammation in the pathogenesis of AD and PD. We have outlined how endogenous triggers, predominantly Aβ, tau, and α-synuclein, activate microglia and astrocytes, linked to inflammatory cascades that may exacerbate neurodegeneration. These glial responses are further modulated by peripheral influences, and the involvement of OLGs and NG2 glia highlights the intricate web of glial crosstalk within neuroinflammatory networks. Exogenous modulators, including systemic inflammation and gut–brain axis signaling, also sculpt CNS immune activity, emphasizing the bidirectional connectivity between central and peripheral inflammatory processes in both disorders.

Nevertheless, several critical areas warrant further investigation. First, the spatiotemporal dynamics of neuroinflammation across distinct brain regions and disease stages in AD and PD remain incompletely characterized. Longitudinal studies employing advanced imaging and multi-omics approaches will be vital for defining stage-specific inflammatory signatures. Second, although microglia and astrocytes have been intensively studied, the distinct contributions of other cell populations, such as endothelial cells, OLGs, and NG2 glia, to the progression and resolution of neuroinflammation require deeper exploration. Third, the interface between central and peripheral inflammation represents an especially promising therapeutic frontier: targeting systemic inflammatory pathways or modulating peripheral organ–brain axes (e.g., gut–brain) may mitigate neuroinflammation and retard disease advancement.

Technological innovation will be central to advancing our understanding and treatment of neuroinflammation in AD and PD. The creation of next-generation biomarkers, especially imaging agents with improved cellular specificity and sensitivity that capture dynamic neuroinflammatory changes, is essential for enabling earlier diagnosis and accurate monitoring of therapeutic response. In parallel, human iPSC–derived models and brain organoids provide powerful platforms for investigating human-specific neuroinflammatory mechanisms and for high-throughput screening of candidate therapeutics.

From a therapeutic perspective, future strategies should strive to rebalance pro-inflammatory and anti-inflammatory pathways rather than indiscriminately suppressing inflammation, given that appropriately regulated inflammatory processes contribute to protein clearance and tissue repair. Existing evidence indicates that neuroinflammation in AD and PD is governed by a highly intricate interplay between central and peripheral systems. Of particular importance, delaying or even reversing age-related dysfunction of glial cells may be critical for restoring equilibrium in neuroinflammatory signaling. Thus, combinatorial approaches that concurrently target central and peripheral inflammatory drivers hold promise for superior efficacy. Identifying key regulatory nodes within neuroinflammatory networks will pave the way for more precise immunomodulation with minimized off-target effects.

In conclusion, neuroinflammation represents a prominent and therapeutically tractable component associated with the pathogenesis of AD and PD. Most current evidence remains associative rather than causally definitive. Neuroinflammation likely acts as a disease amplifier in most contexts, whereas its role as a primary disease initiator still requires further clinical validation. Advancing the field will demand a truly multidisciplinary framework integrating neuroscience, geriatrics, genetics, immunology, neurology, and cutting-edge technology to translate current mechanistic insights into effective interventions. Future research should prioritize delineating the context-specific functions of glial cell subsets, validating novel biomarkers, and developing targeted immunomodulatory therapies that may alter the progressive trajectory of these devastating neurodegenerative disorders.

## Data Availability

No datasets were generated or analysed during the current study.

## Abbreviations

AD: Alzheimer’s disease
PD: Parkinson’s disease
Aβ: Amyloid-β
OPCs: Oligodendrocyte precursor cells
OLGs: Oligodendrocytes
iPSC: Induced pluripotent stem cell
SNpc: Substantia nigra pars compacta
CNS: Central nervous system
IL-1β: Interleukin-1β
TNF-α: Tumor necrosis factor-α
BBB: Blood-brain barrier
APP: Amyloid precursor protein
LPS: Lipopolysaccharide
DAM: Disease-associated microglia
TLR2: Toll like receptor 2
NF-κB: Nuclear factor-κB
NLRP3: NOD-like receptor family pyrin domain containing 3
CSF: Cerebrospinal fluid
MMD: Mild motor deficit
IFN-γ: Interferon-γ
IBD: Inflammatory bowel disease
FMT: Fecal microbiota transplantation
cPLA2: Cytosolic phospholipase A2
MAGL: Monoacylglycerol lipase
MPTP: 1-methyl-4-phenyl-1,2,3,6-tetrahydropyridine
ASM: Acid sphingomyelinase
RAGE: Receptors for advanced glycation end products
cGAS: Cyclic GMP–AMP synthase
STING: Stimulator of interferon genes
DHA: Docosahexaenoic acid
NPD1: Neuroprotectin D1
APOE4: ε4 allele of apolipoprotein E
ERK: Extracellular signal-regulated kinase
CX3CR1: CX3C chemokine receptor 1
PSAP: Prosaposin
NK cells: Natural killer cells
Treg: Regulatory T
LRRK2: Leucine-rich repeat kinase 2
MCI: Mild cognitive impairment
OxPS: Oxidized phosphatidylserine
LPC: Lysophosphatidylcholine
PET: Positron emission tomography
TSPO: Translocator protein
RNA‑seq: RNA sequencing
scRNA‑seq: Single‑cell RNA sequencing
snRNA‑seq: Single‑nucleus RNA sequencing
HBOs: Human brain organoids
ARIA: Amyloid-related imaging abnormalities
PPARγ: Peroxisome proliferator-activated receptor-gamma
ATV: antibody transport vehicle
